# Supplementary material for: A self-healing plastic ceramic electrolyte by an aprotic dynamic polymer network for lithium metal batteries
Source: Nat Commun. 2024 Nov 19;15:10015. doi: 10.1038/s41467-024-53869-z (PMC11576998; doi:10.1038/s41467-024-53869-z)
Supplement: Supplementary file 1 — Supplementary Information [file 41467_2024_53869_MOESM1_ESM.pdf]

Supplementary materials for

A self-healing plastic ceramic electrolyte by an aprotic dynamic polymer network for lithium metal batteries

Yubin He[1,||], Chunyang Wang[1,||], Rui Zhang[1], Peichao Zou[1], Zhouyi Chen[1], Seong-Min Bak[2], Stephen E. Trask[3], Yonghua Du[2], Ruoqian Lin[4], Enyuan Hu[5], Huolin L. Xin[1,*]

[1]Department of Physics and Astronomy, University of California, Irvine, CA, USA.
[2]National Synchrotron Light Source II, Brookhaven National Laboratory, Upton, NY, 11973, USA
[3]Cell Analysis, Modeling, and Prototyping Facility, Argonne National Laboratory, Lemont, Illinois, 60439, USA
[4]Department of Mechanical Engineering, University of California, Riverside, CA
[5]Chemistry Division, Brookhaven National Laboratory, Upton, NY, 11973, USA.

[*]Correspondence should be addressed to huolin.xin@uci.edu (H.L.X.).
[||]These authors contributed equally to this work.

This file includes:
Supplementary Figure S1-S38
Supplementary Table S1-S3
Supplementary References

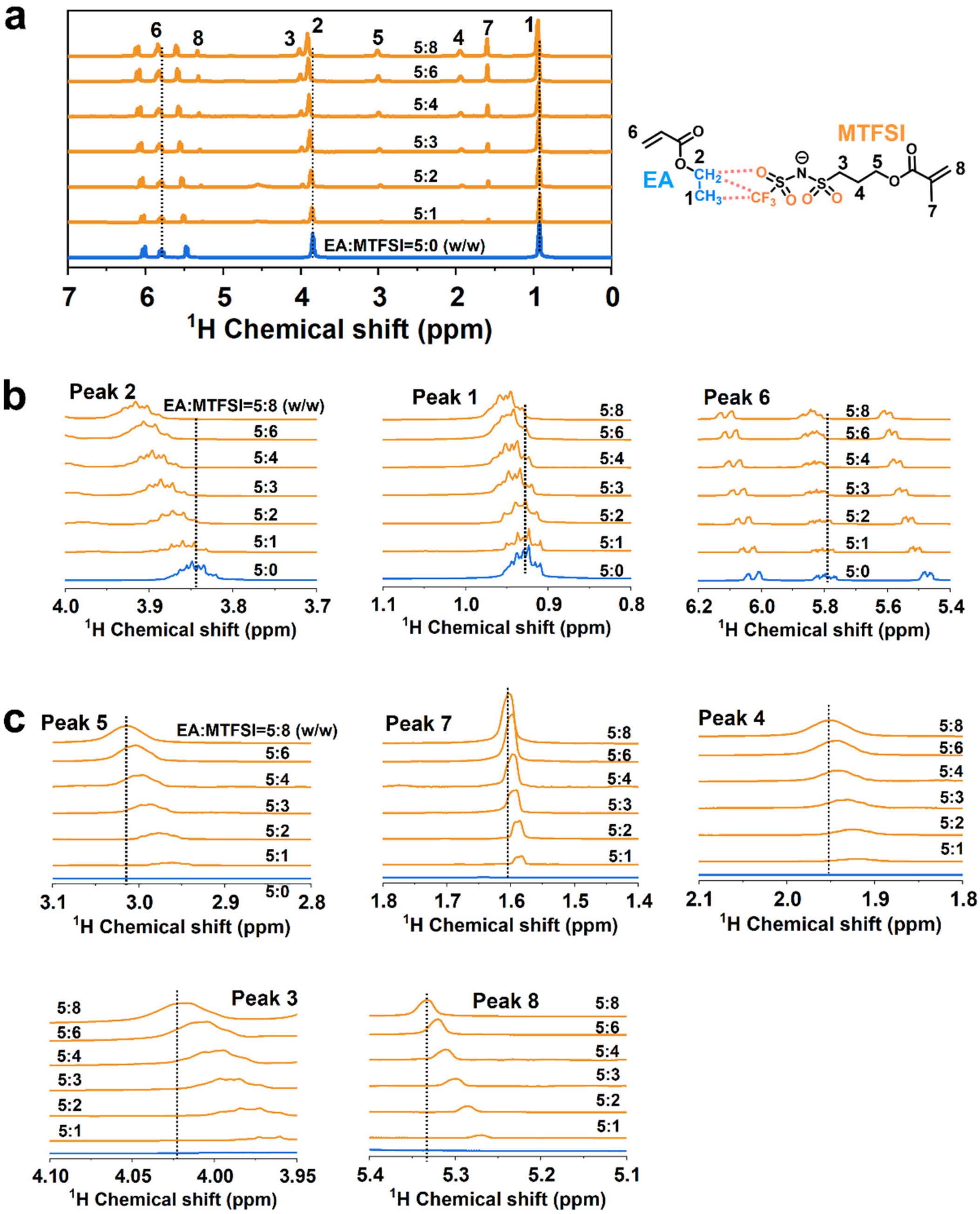

Figure S1. a. Full range Magic Angle Spinning Solid-State NMR (MAS-ssNMR) spectra of EA/MTFSI mixture at different mass ratio. The spectra were referenced against trimethylsilane (TMS, 0 ppm). The spinning rate of sample is 2000 Hz. **b.** Enlarged MAS-ssNMR spectra showing the downfield shifting of EA signals with increasing MTFTSI content. **c.** Enlarged MAS-ssNMR spectra showing the upfield shifting of MTFSI signals with increasing EA content.

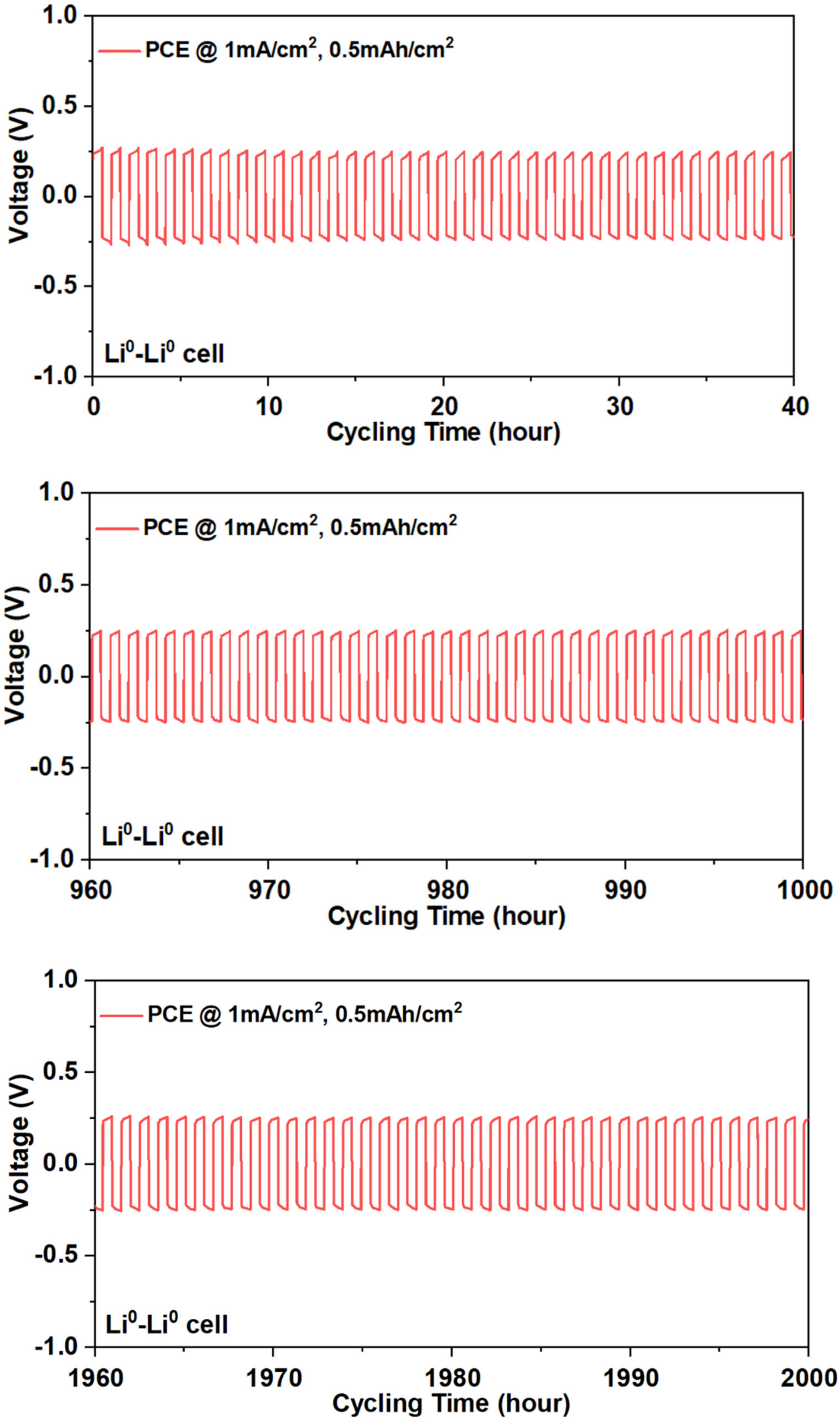

Figure S2. Enlarged voltage-time profiles as a supplement to Li^0|PCE|Li^0 cell performance shown in Figure 1e. The cell was cycled at $1mA/cm^2$, $0.5mAh/cm^2$, and r.t. The cell was rested for 60s after charging and discharging for 0.5hr.

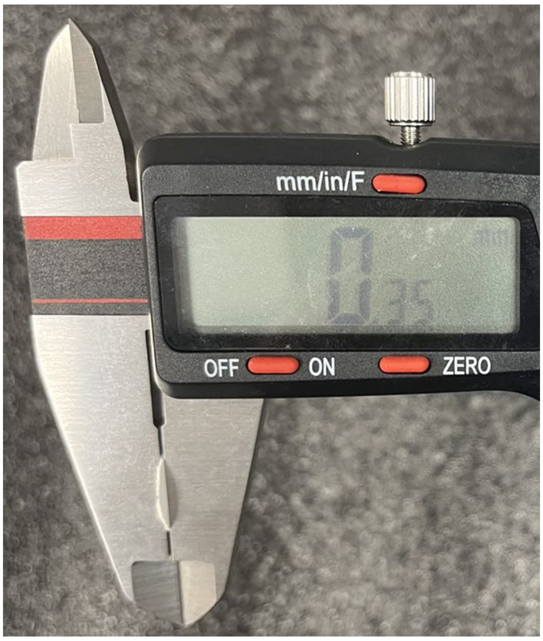

Figure S3. Photographs showing the thickness of PCE film is 350 μm.

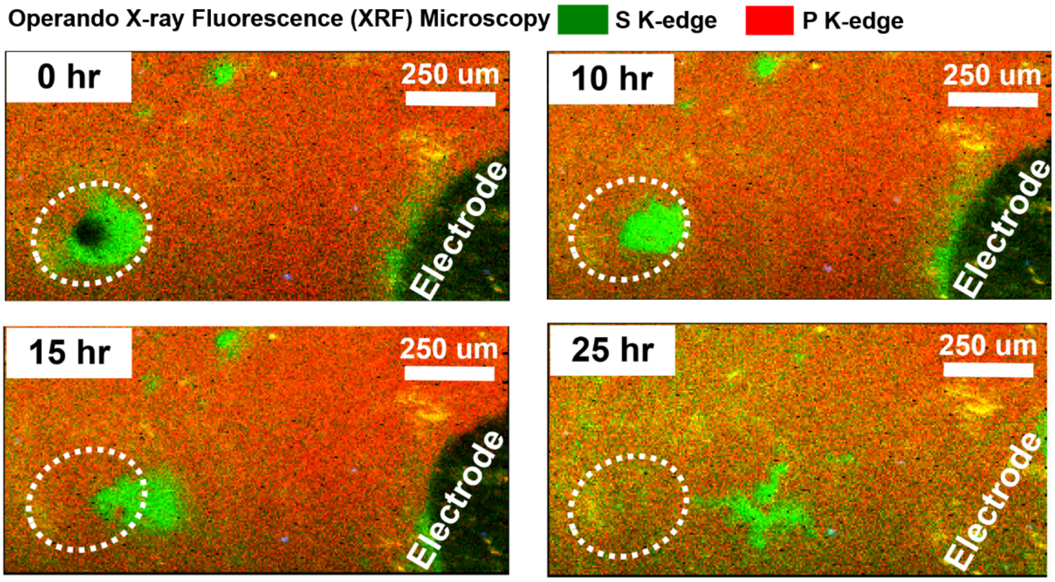

Figure S4. High-resolution XRF images at the PCE-electrode interface showing the excellent wettability and interface compatibility of PCE towards the electrode. As illustrated by the tube geometry of the *in-situ* cell (Figure 2b), the SS electrode's diameter is slightly smaller than the inner diameter of the Kapton® tube, allowing for its insertion into the tube. This creates a small gap between the SS electrode and the Kapton® tube. Over cycling time, PCE infiltrates this gap in a manner similar to its infiltration into voids during the self-healing process. Consequently, the electrode becomes obscured by the infiltrated PCE. This observation suggests good wettability and interface compatibility of the PCE with the electrode.

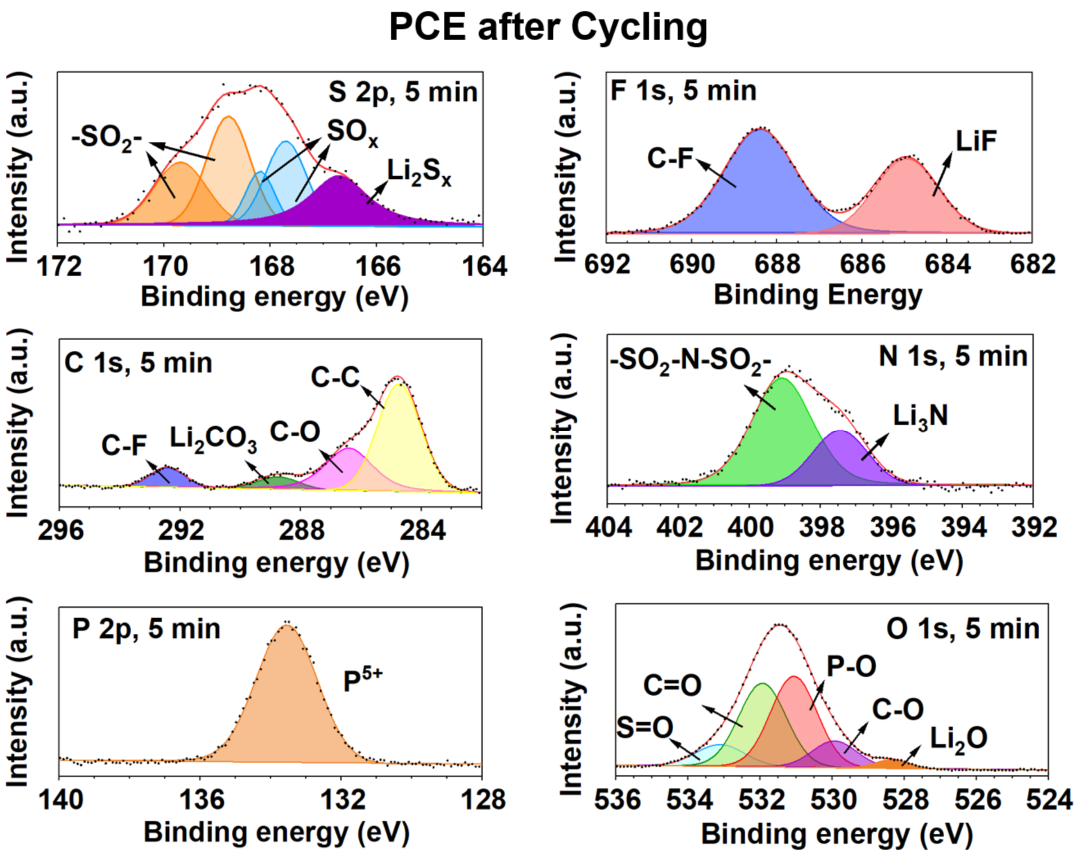

Figure S5. S *2p*, F *1s*, C *1s*, N *1s*, P *2p*, and O *1s* XPS profiles of the PCE derived SEI after Ar sputtering for 5 minutes. SO_x, Li_2S_x, LiF, Li_2O, Li_3N, and Li_2CO_3 in SEI were mainly originated from the electrochemical reduction of SH-SPE, while P *2p* and O *1s* profiles suggest the successful protection of LATP from degradation.

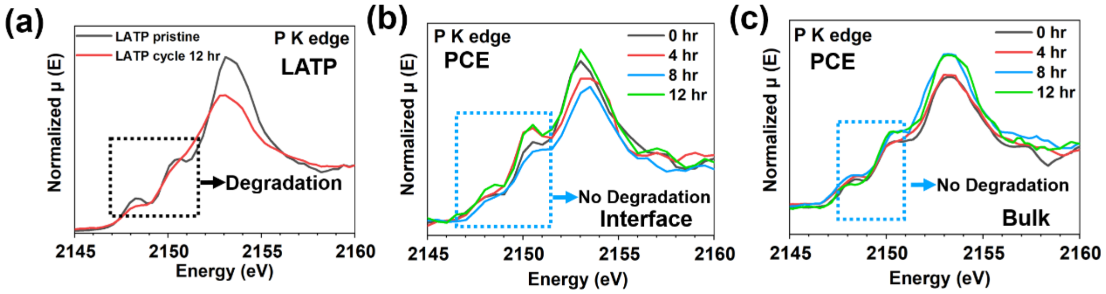

Figure S6. (a) Ex-situ P K-edge XAS of LATP before and after cycling in a Li^0-Li^0 symmetric cell; **(b, c)** Operando P K-edge XAS obtained at PCE-electrode interface **(b)** and bulk PCE area **(c)**.

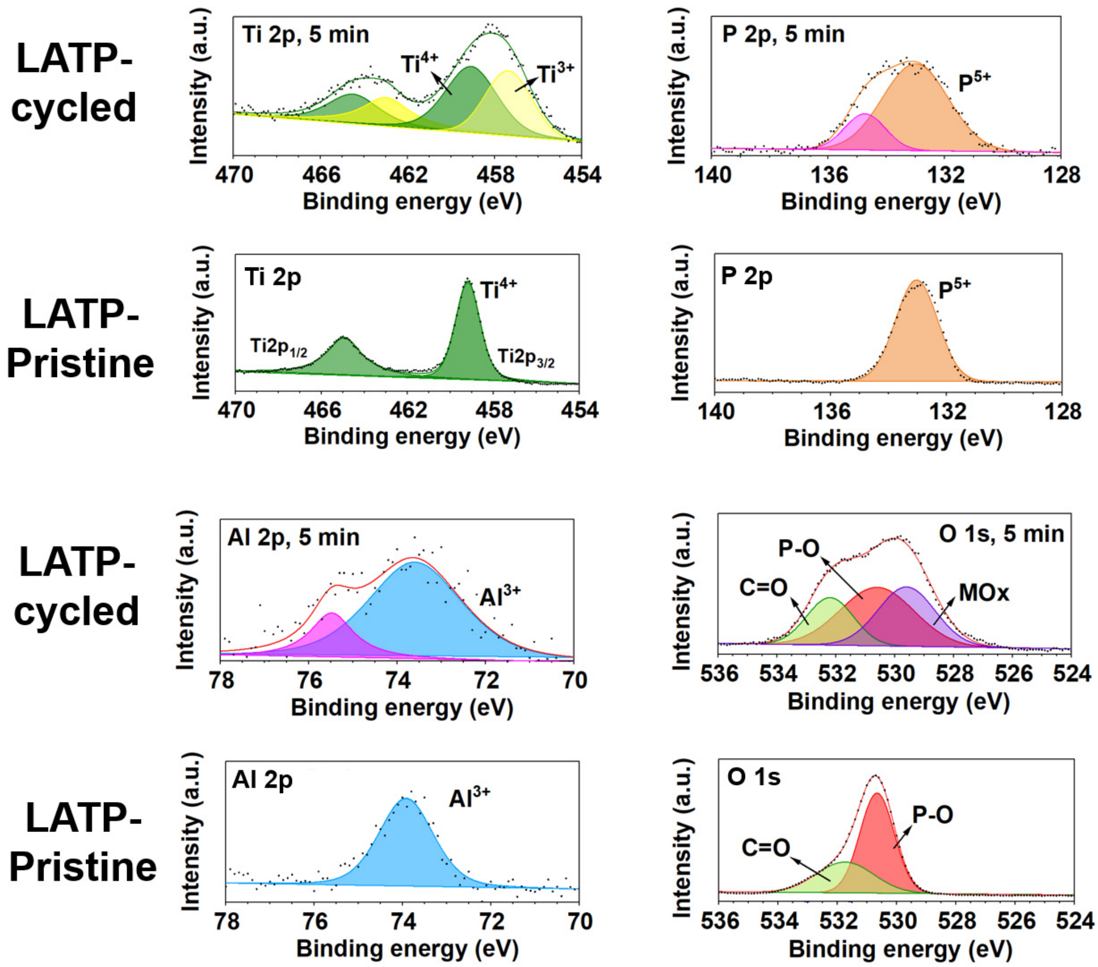

Figure S7. Ti *2p*, P *2p*, Al *2p*, and O *1s* XPS profiles of pristine LATP, and LATP-derived-SEI after cycling in a Li^0-Li^0 symmetric cell. The emergence of reduced P signal and MO_x signal suggest the degradation of LATP.

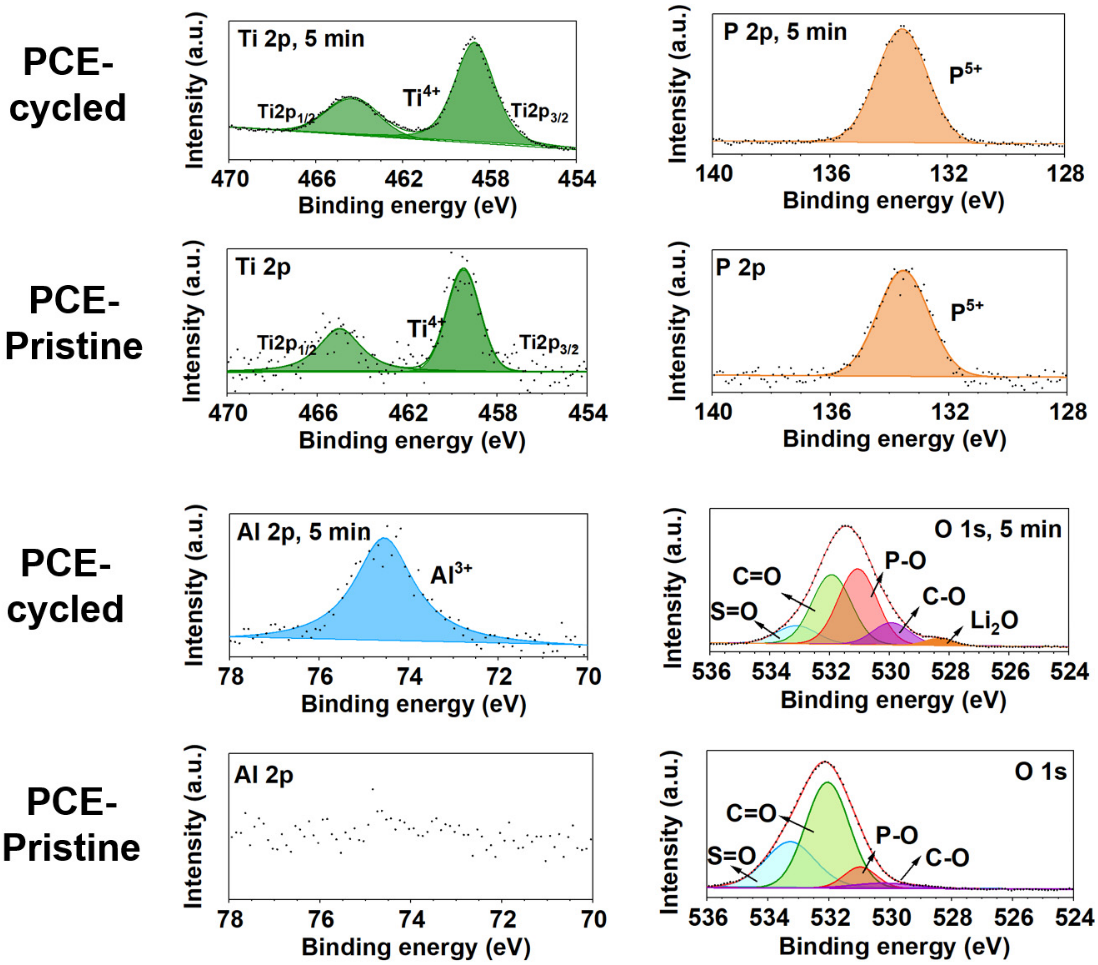

Figure S8. Ti *2p*, P *2p*, Al *2p*, and O *1s* XPS profiles of pristine PCE, and PCE-derived-SEI after cycling in a Li^0-Li^0 symmetric cell. Ti, P, and Al signals remain unchanged because the SH-SPE-derived SEI prevent the degradation of LATP.

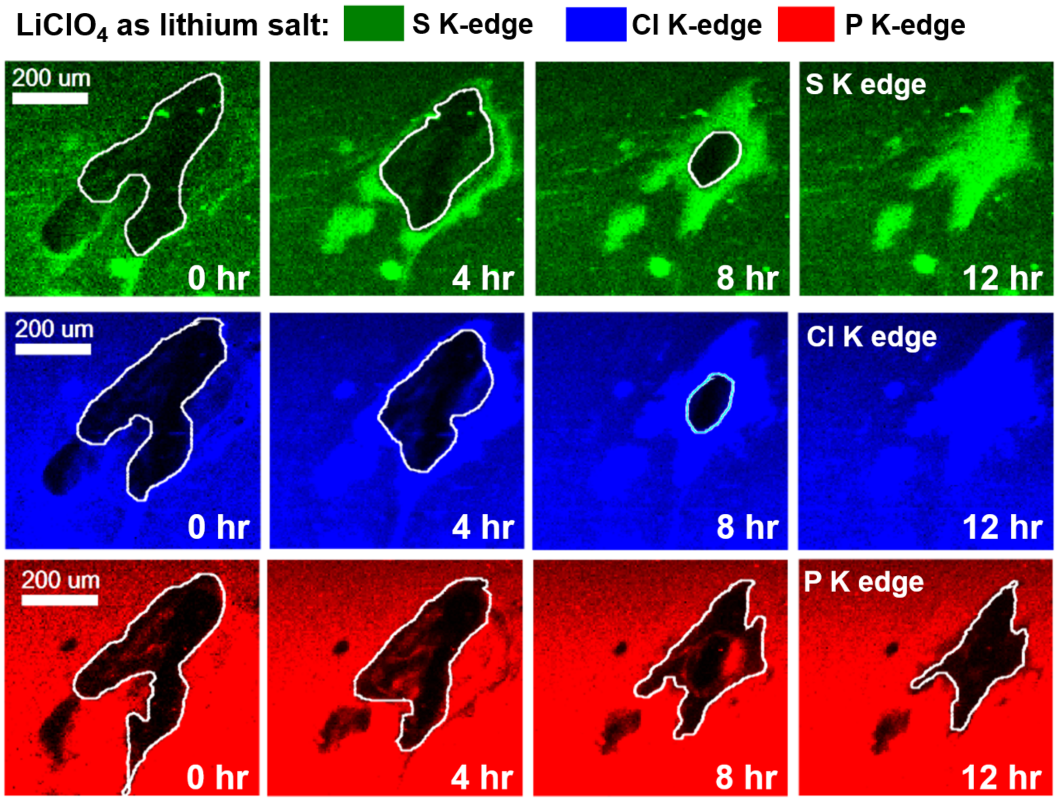

Figure S9. Individual S, Cl, and P K-edge XRF mapping showing the migration of polymer mainchain (S), lithium salt (Cl), and LATP (P).

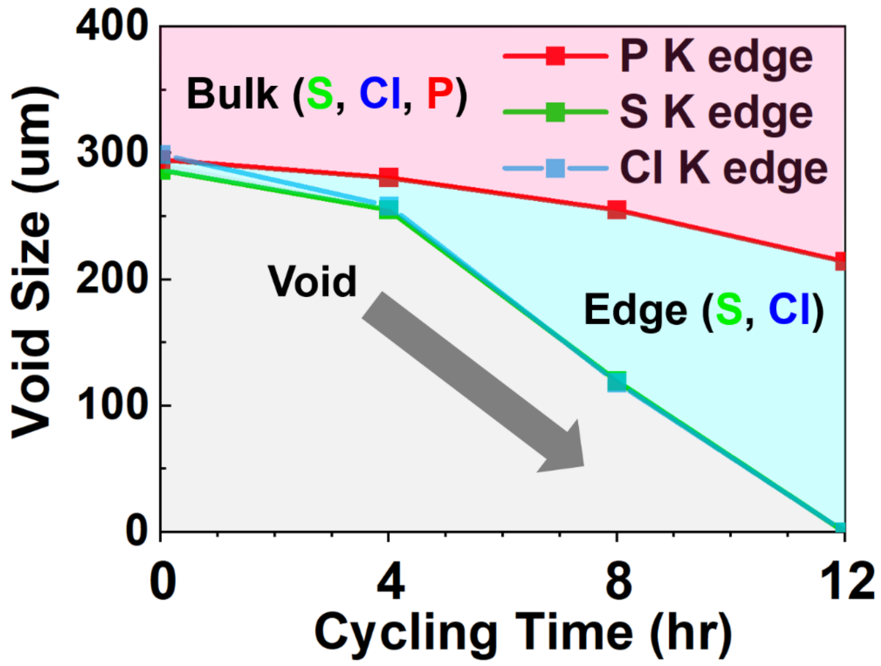

Figure S10. Calculated void size evolution based on the P, S, and Cl mapping.

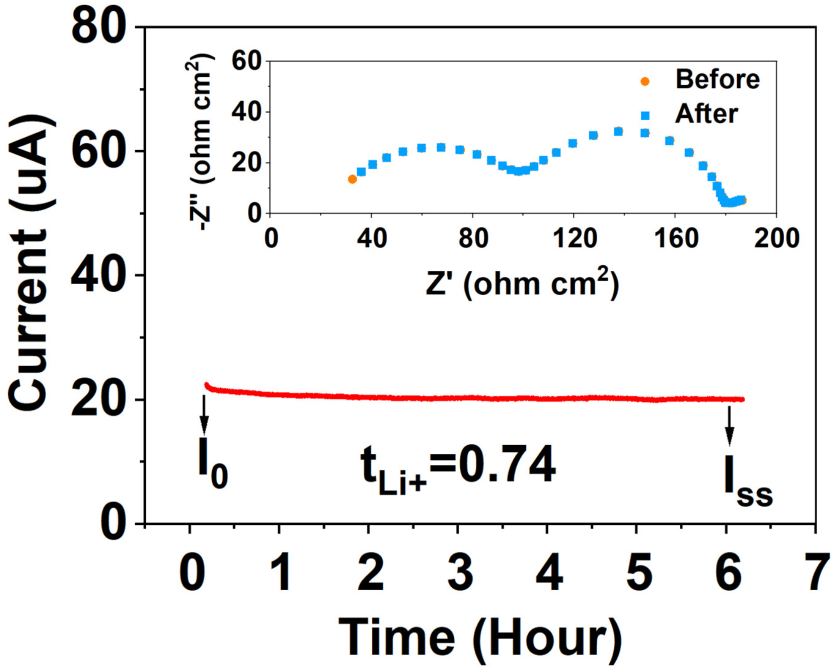

Figure S11. Li^+ transference number (t_{Li+}) of PCE measured under a Li^0-Li^0 symmetric cell configuration and r.t.

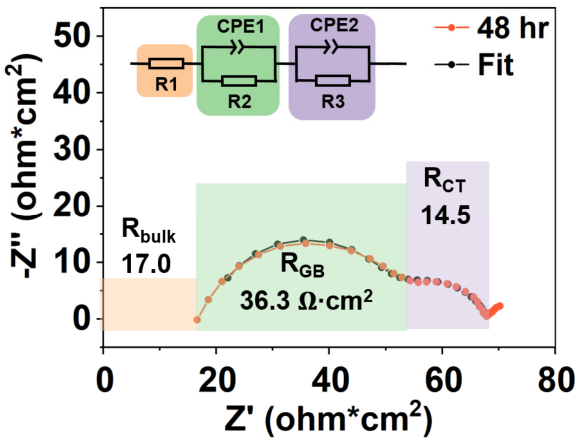

	R_b	R_{gb}	R_{CT}
Value (ohm*cm²)	17.0	36.3	14.5
Error (%)	2.1	4.6	9.2

Figure S12. Equivalent circuit fitting for EIS plot of Li^0|PCE|Li^0 cells after cycling at 0.2 mA/cm^2 for 48 hours. R_b, R_{gb}, and R_{CT} are bulk resistance, grain boundary resistance, and charge transfer resistance, respectively.

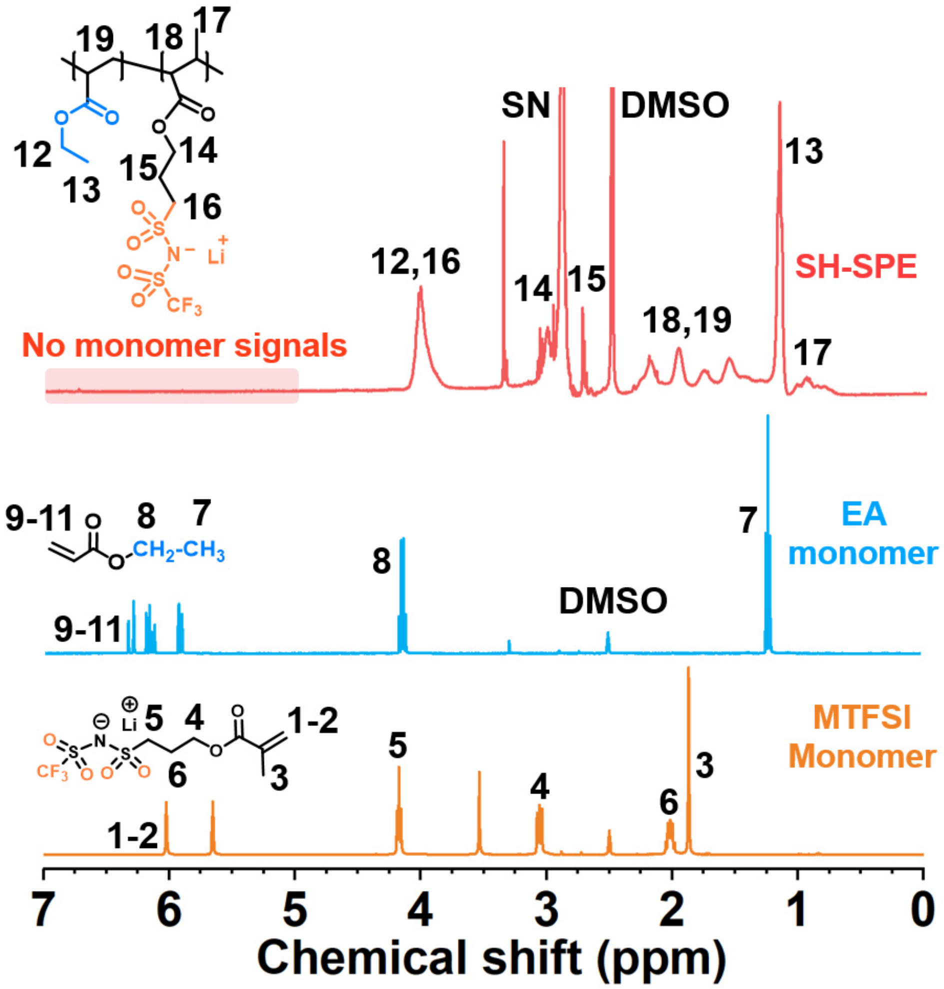

Figure S13. ^{1}H NMR spectra of EA, MTFSI, and SH-SPE showing the monomer conversion yield and polymer structure of SH-SPE.

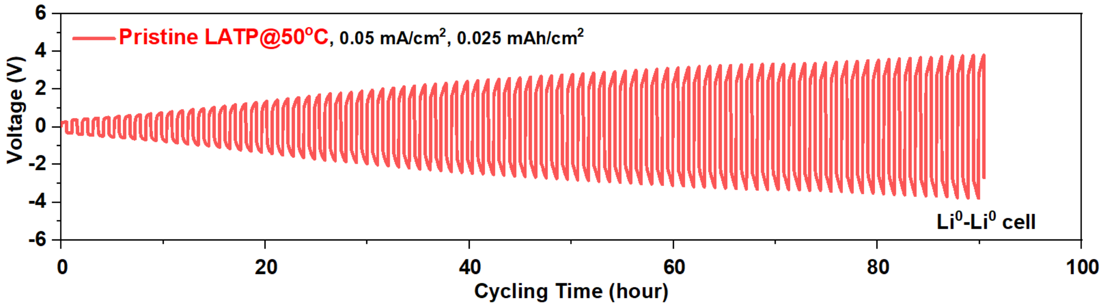

Figure S14. Voltage-time profiles at 0-100 hours of solid-state Li^0-Li^0 cells employing **pristine LATP** as electrolyte. The Li^0|LATP|Li^0 before 100 hour due to rapid overpotential build-up caused by uncontrolled side reactions.

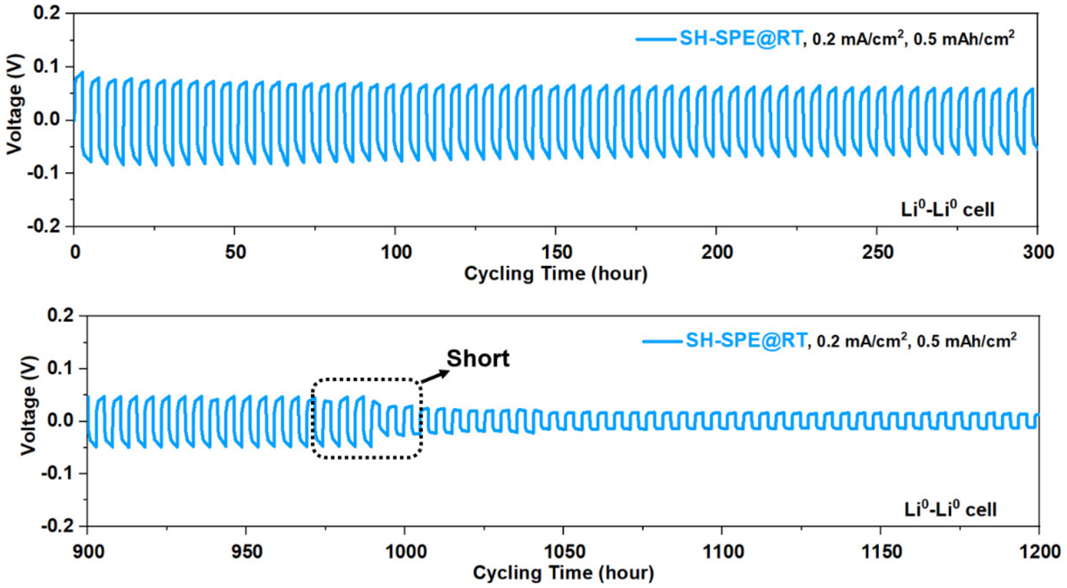

Figure S15. Enlarged voltage-time profiles as a supplement to Li^0|**SH-SPE**|Li^0 cell performance shown in Figure 5a. The cell was cycled at $0.2mA/cm^2$, $0.5mAh/cm^2$, and r.t. The cell was rested for 5mins after charging and discharging for 2.5hr.

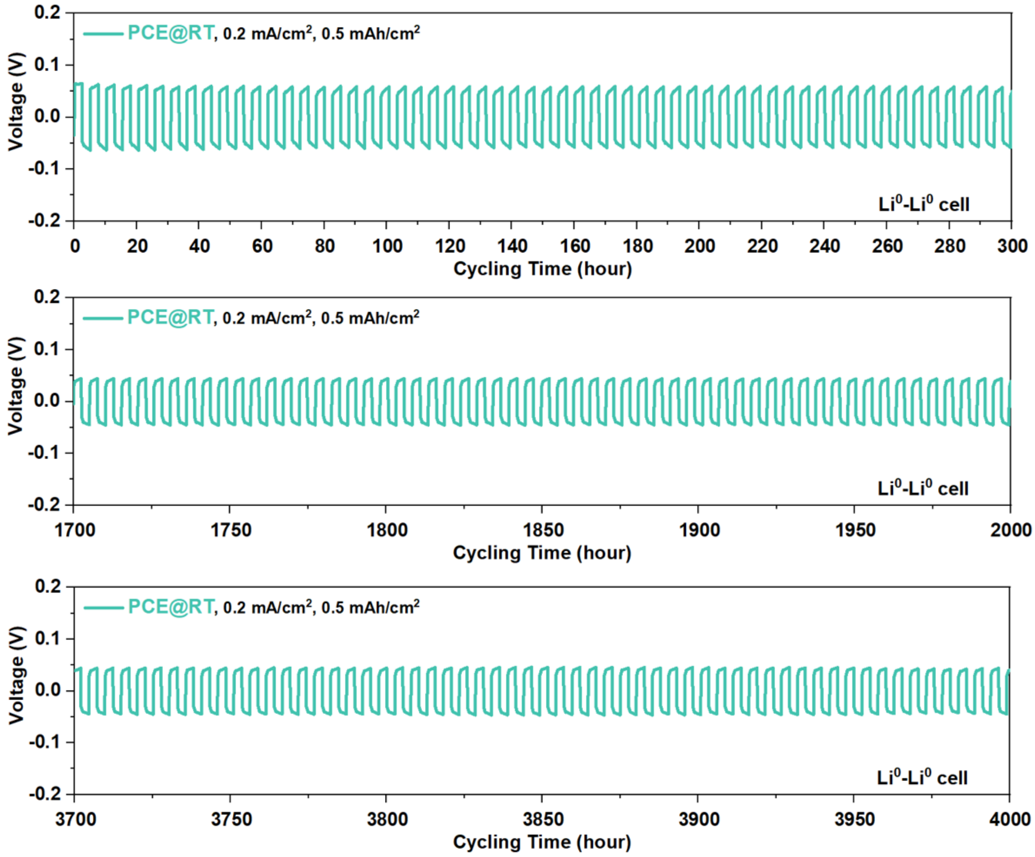

Figure S16. Enlarged voltage-time profiles as a supplement to Li^0|**PCE**|Li^0 cell performance shown in Figure 5a. The cell was cycled at $0.2mA/cm^2$, $0.5mAh/cm^2$, and r.t. The cell was rested for 5mins after charging and discharging for 2.5hr.

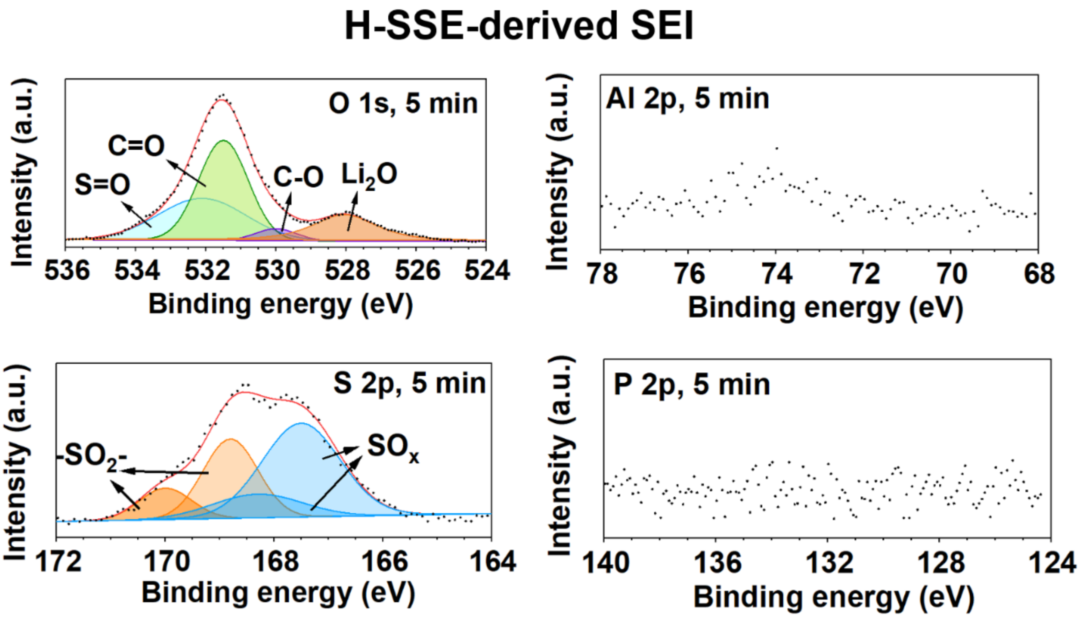

Figure S17. O *1s*, S *2p*, P *2p*, and Al *2p* XPS profiles of the H-SSE-derived-SEI after Ar sputtering for 5 minutes. The absence of Ti, P, and Al elements in the SEI suggests complete isolation of LATP from Li^0 anode by the PA-SPE buffer layer.

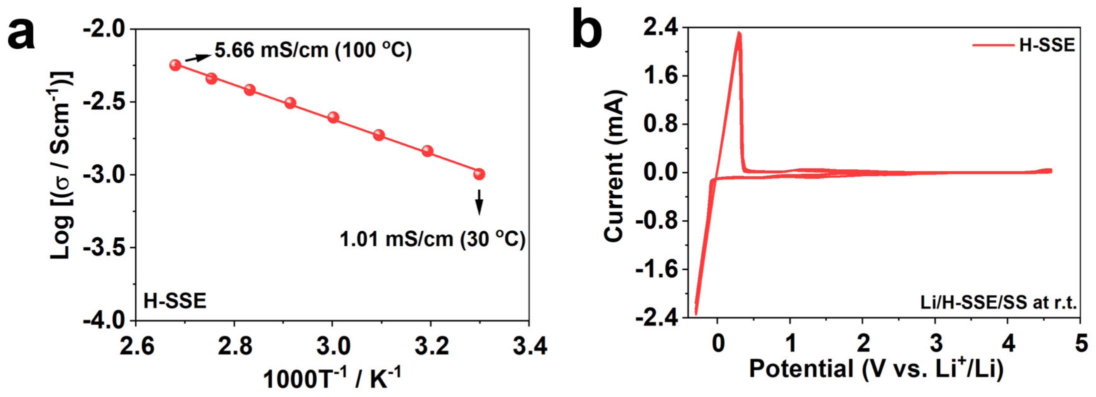

Figure S18. (a) Temperature-dependent conductivity of H-SSE and the Arrhenius fitting in the form of $\sigma = Ae^{-\frac{E_a}{kT}}$. **(b)** Cyclic voltammetry (CV) of the Li|H-SSE|SS cell at r.t. and 1mV/s showing the electrochemical stability window of H-SSE.

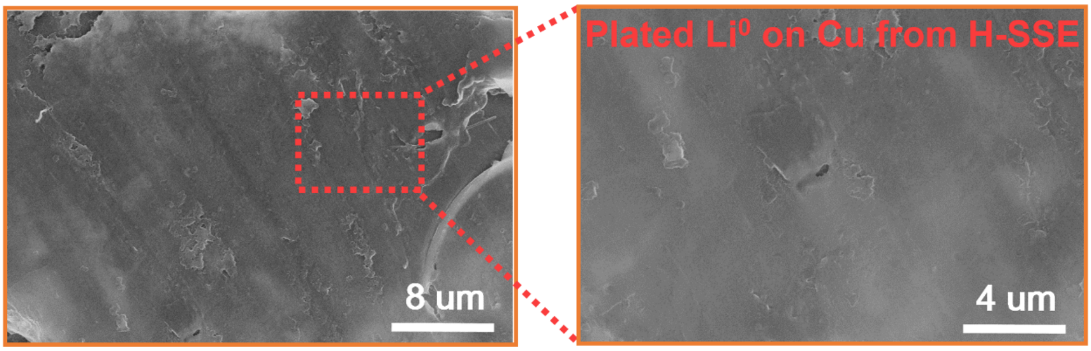

Figure S19. SEM images showing the surface morphology of Li^0 deposits after discharging a Li^0|H-SSE|Cu cell at 0.2 mA/cm^2 for 10 hours at r.t.

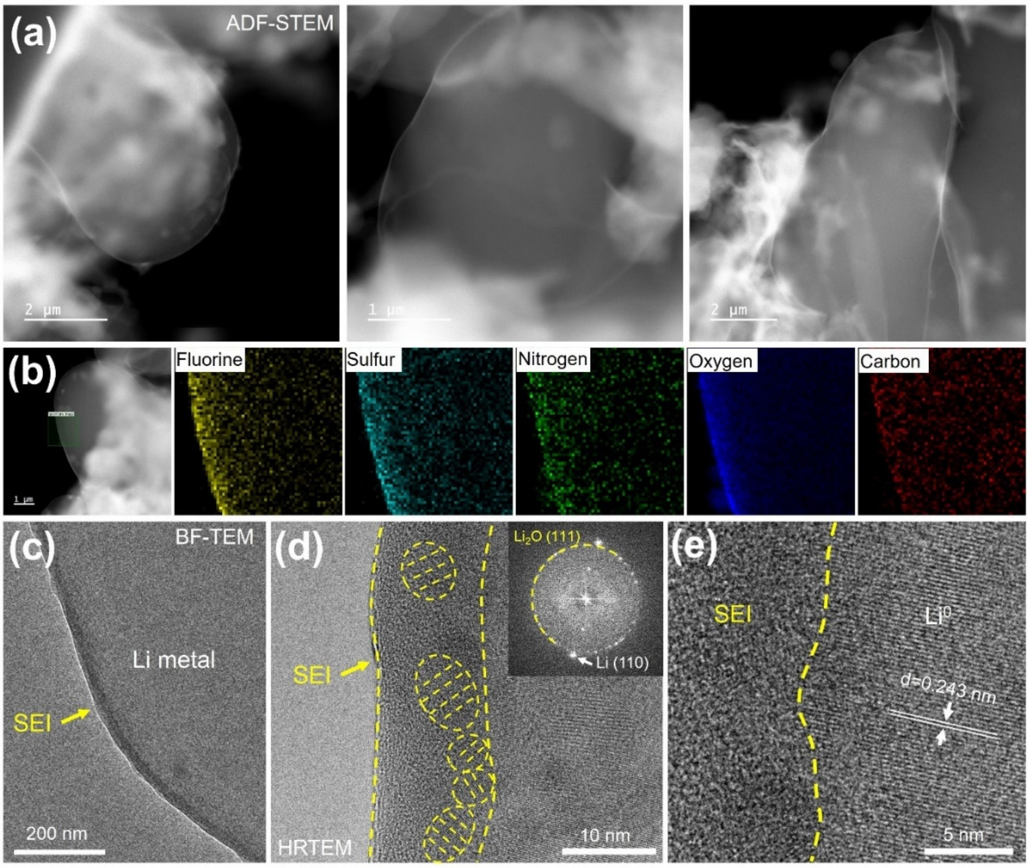

Figure S20. Cryo-TEM images showing the morphology of Li^0 deposited under H-SSE. (a) Representative cryogenic transmission electron microscopy (Cryo-TEM) images showing the smooth chunk morphology of the deposited Li^0 with H-SSE. The image was obtained in high-angle annular dark-field (HAADF) mode; **(b)** High-angle annular dark-field STEM (HAADF-STEM) image, and energy-dispersive spectroscopic (EDS) maps of the deposited Li^0 with the H-SSE electrolyte. The result shows that a thin and uniform layer of solid electrolyte interface (SEI) enriched in C, N, O, F, and S forms on the Li^0 surface; **(c)** High-magnification bright-field TEM (BF-TEM) image showing the uniform SEI formed on the Li^0 surface; **(d)** Atomic-resolution Cryo-STEM image showing the structure of the Li^0 deposit and SEI. The SEI comprises nano-sized domains (e.g., Li_2O indicated by

dash circles) with varied crystallographic orientations. The Bragg spots and polycrystalline ring in the fast Fourier transform (FFT) correspond to the Li^0 (110) and Li_2O (111) crystallographic planes, respectively; **(e)** Enlarged image showing the interface between the SEI and the Li^0 deposit. The (110) plane corresponding to Li^0 is identified in the image.

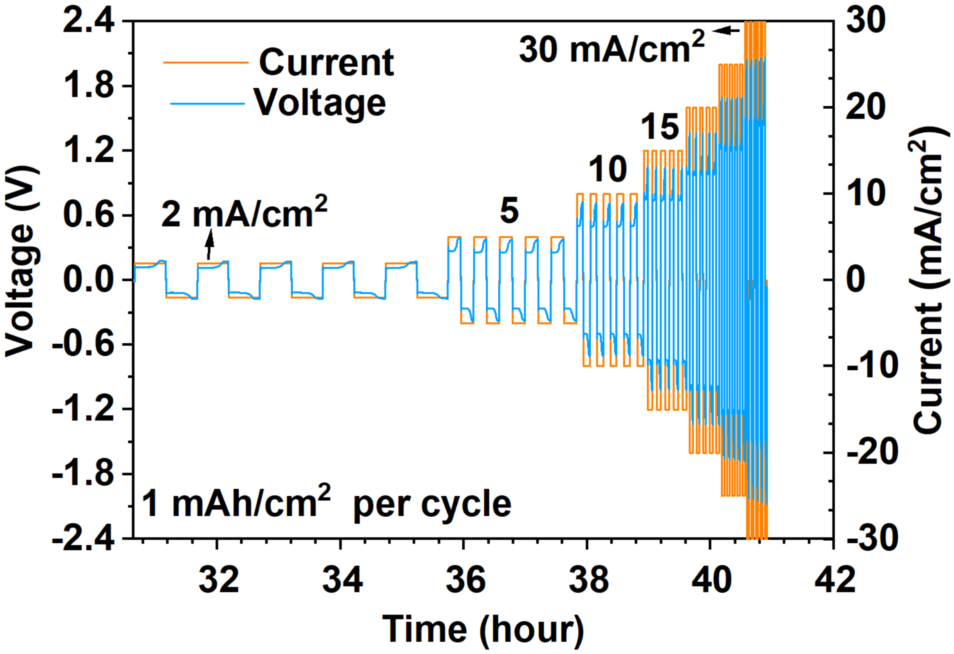

Figure S21. The critical current density of Li^0|H-SSE|Li^0 cell measured at step-up current densities and areal capacities of 1 mAh/cm^2. The Voltage-time curve at lower current densities (0.2-1mA/cm^2) was shown in Figure S22. The testing temperature is 50 °C.

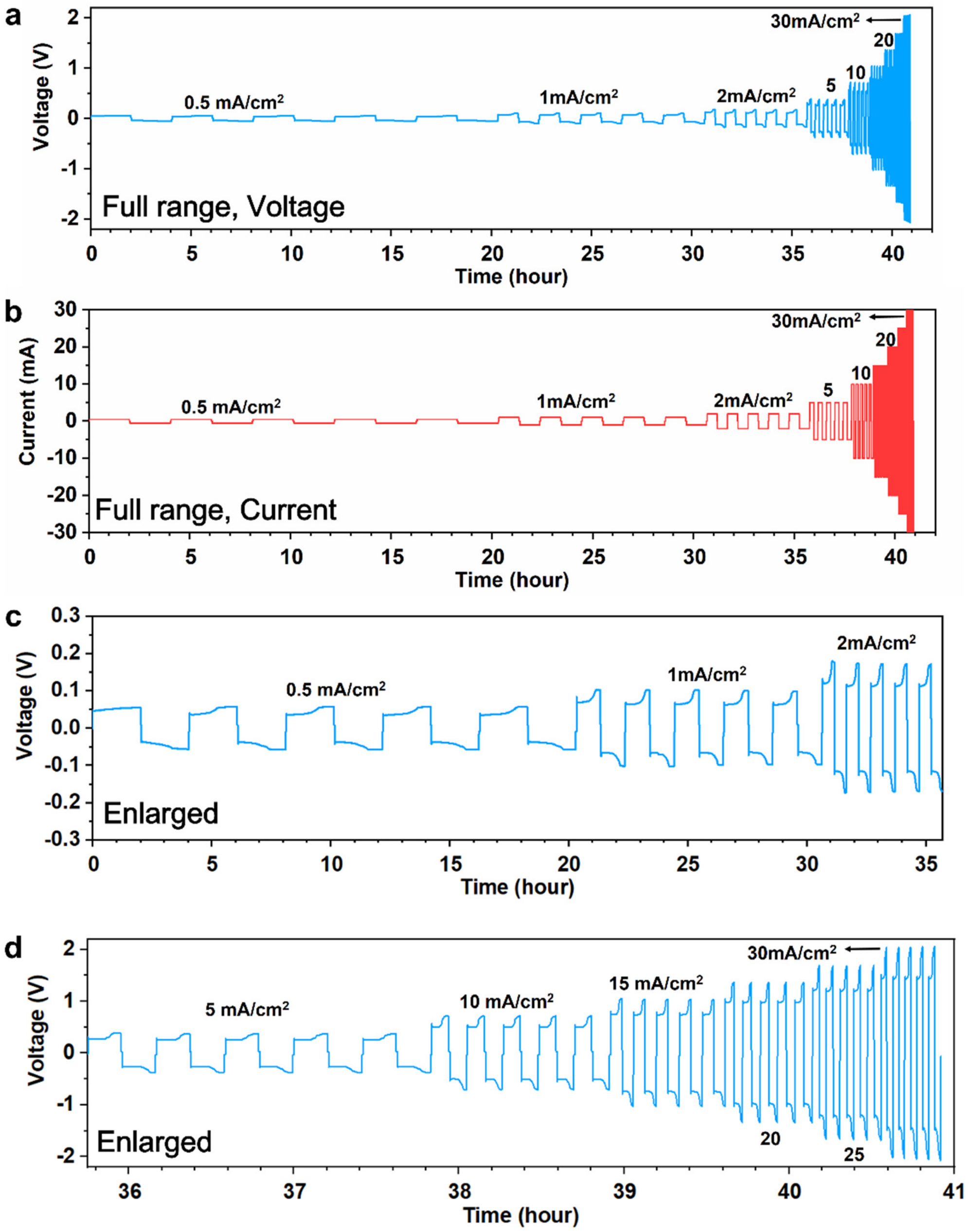

Figure S22. Supplementary enlarged voltage-time profiles showing the critical current density of Li^0|H-SSE|Li^0 cell.

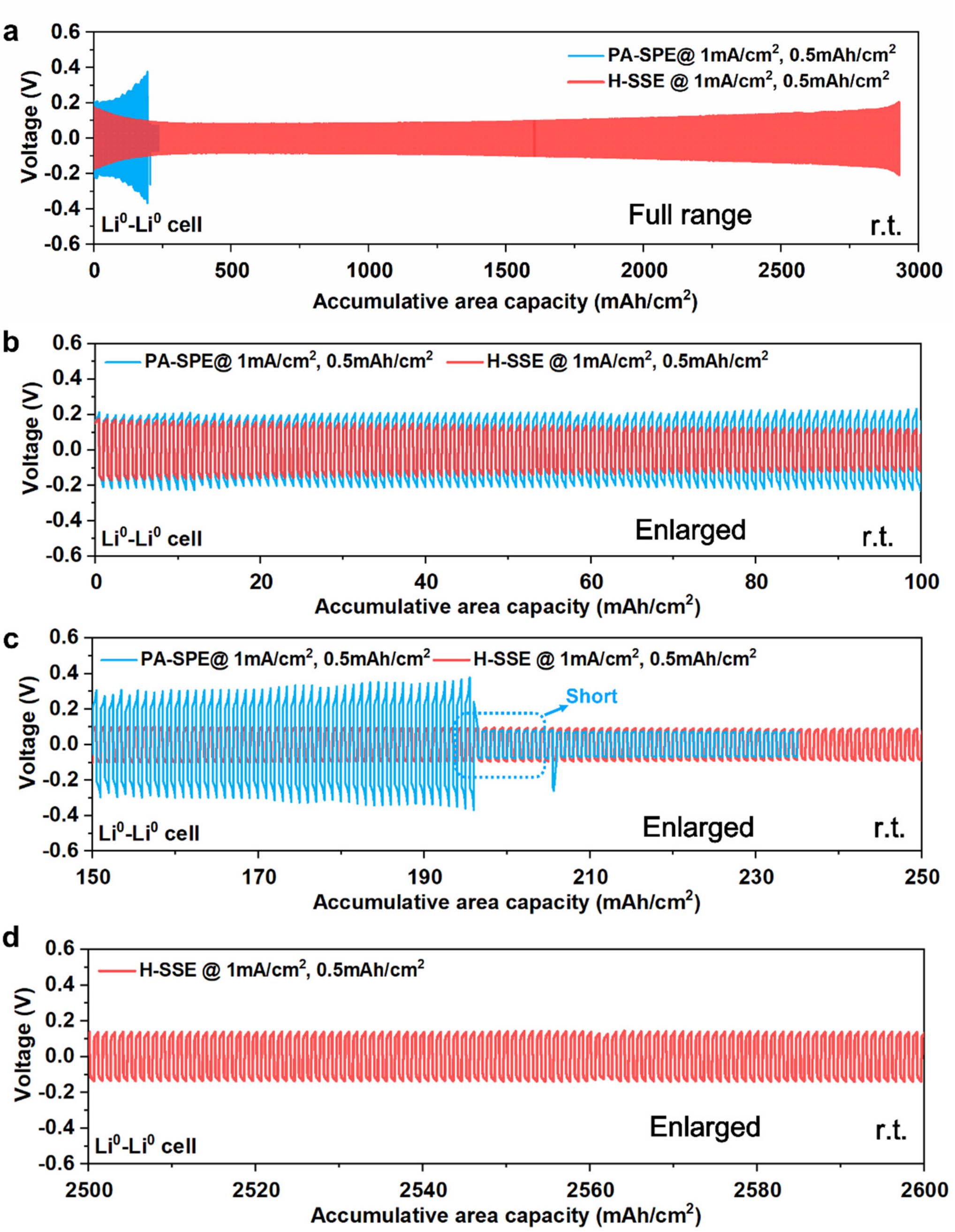

Figure S23. a, Durability comparison of Li^0-Li^0 cells based on H-SSE and PA-SPE at **1mA/cm^2, 0.5mAh/cm^2**. **b, c, d**, Supplementary enlarged profiles. The cell was rested for 3mins after charging and discharging for 0.5hr.

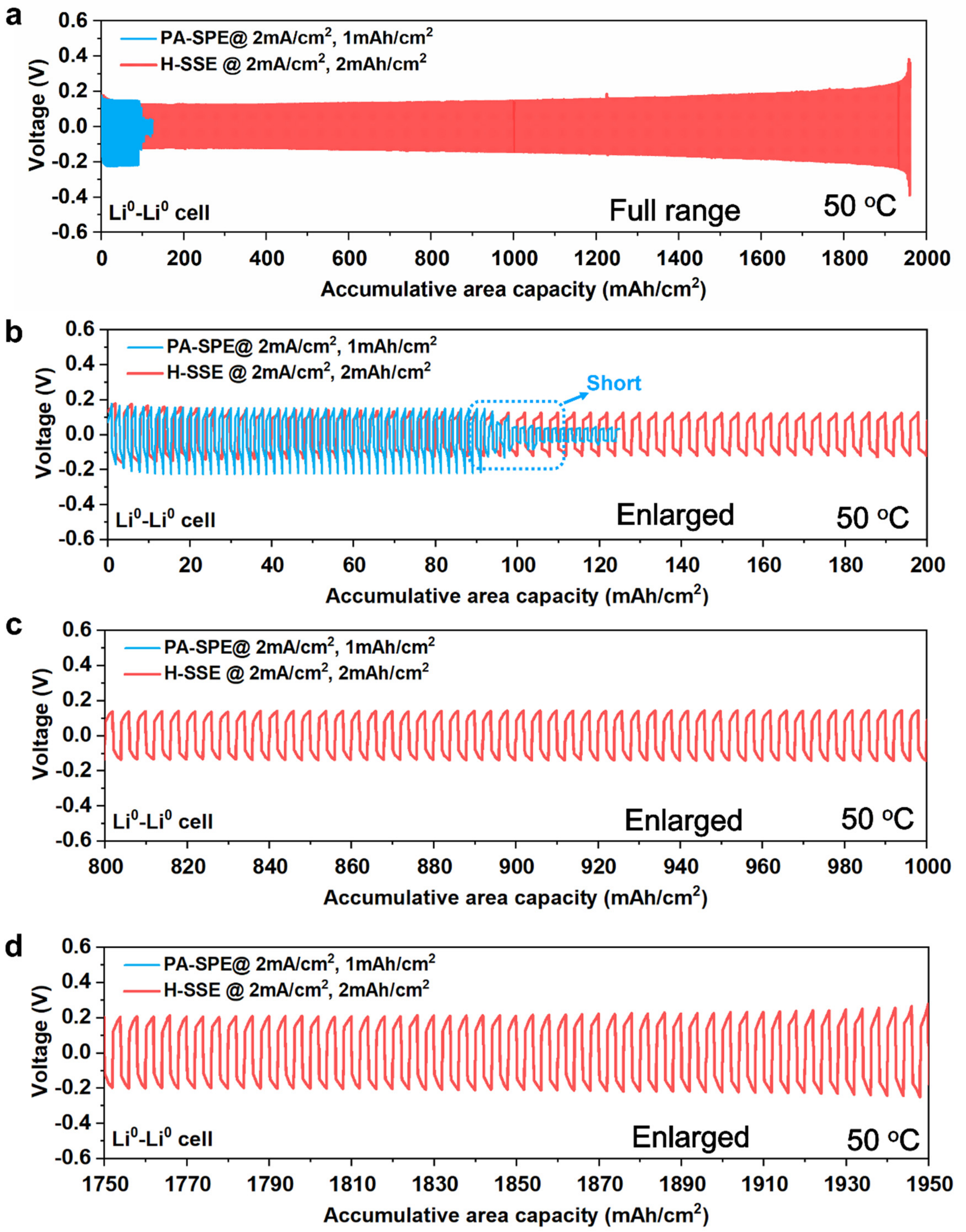

Figure S24. a, Durability comparison of Li^0-Li^0 cells based on H-SSE and PA-SPE at **2mA/cm². b, c, d**, Supplementary enlarged profiles. The cell was rested for 5mins after charging and discharging for 1hr.

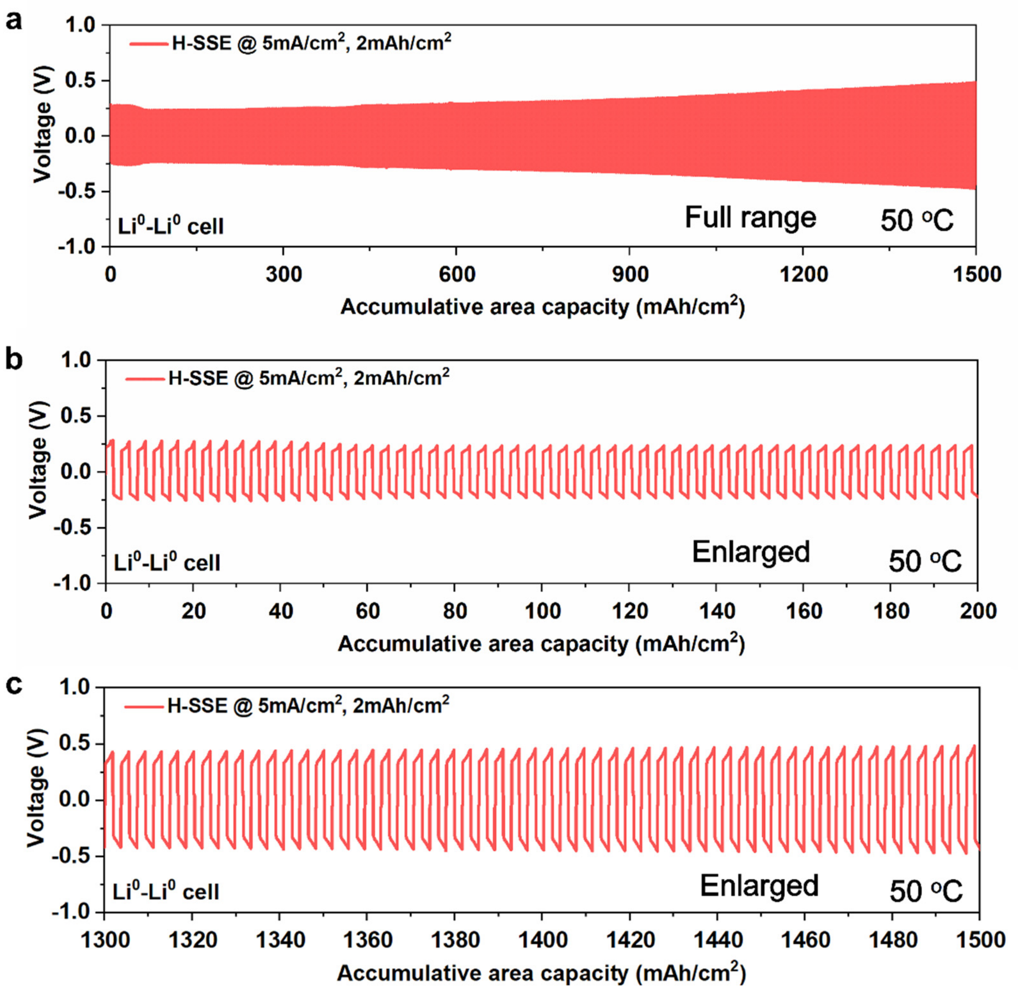

Figure S25. a, Durability of Li^0|H-SSE|Li^0 cells at **5mA/cm^2, 2mAh/cm^2. b, c**, Supplementary enlarged profiles. The cell was rested for 2mins after charging and discharging for 0.4hr.

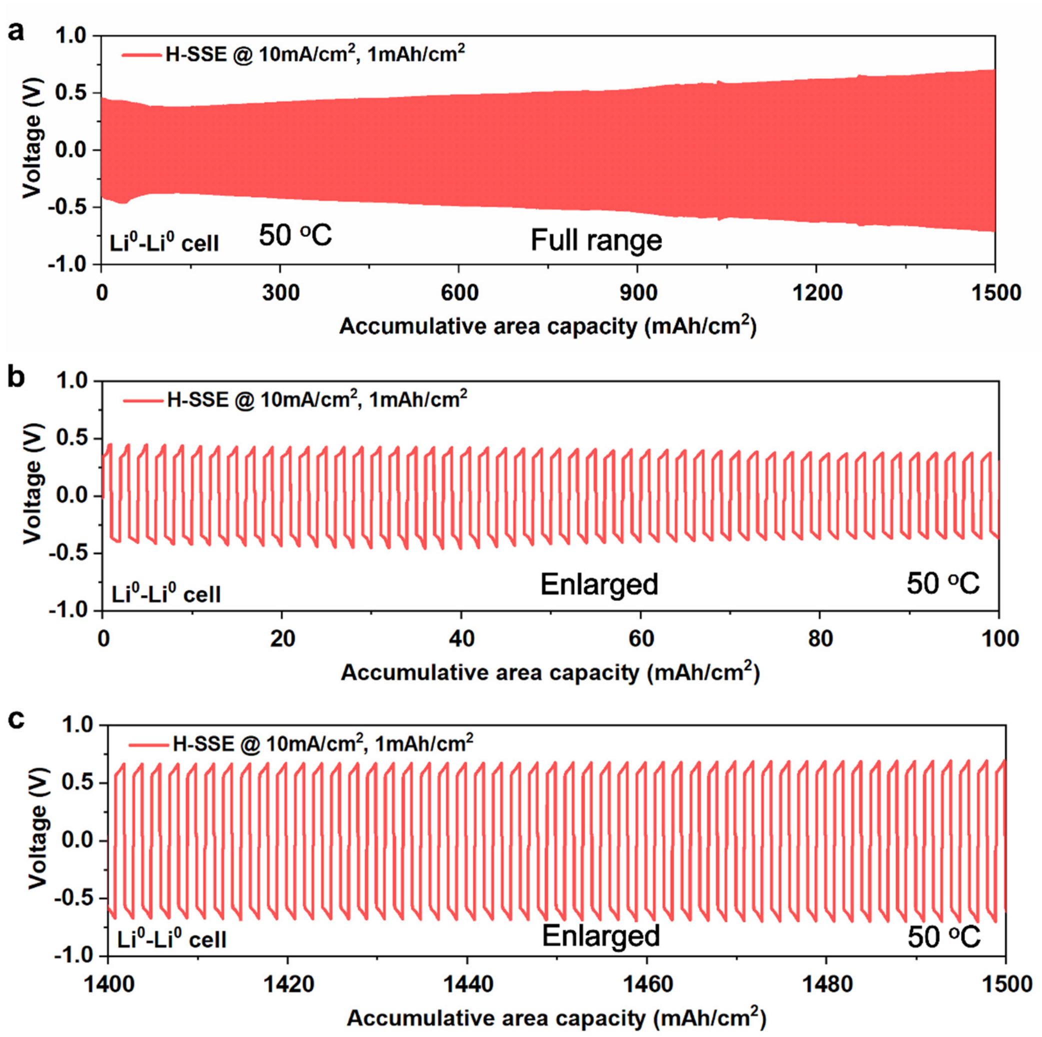

Figure S26. a, Durability of Li^0|H-SSE|Li^0 cells at **10mA/cm^2, 1mAh/cm^2. b, c**, Supplementary enlarged profiles. The cell was rested for 30s after charging and discharging for 0.1hr.

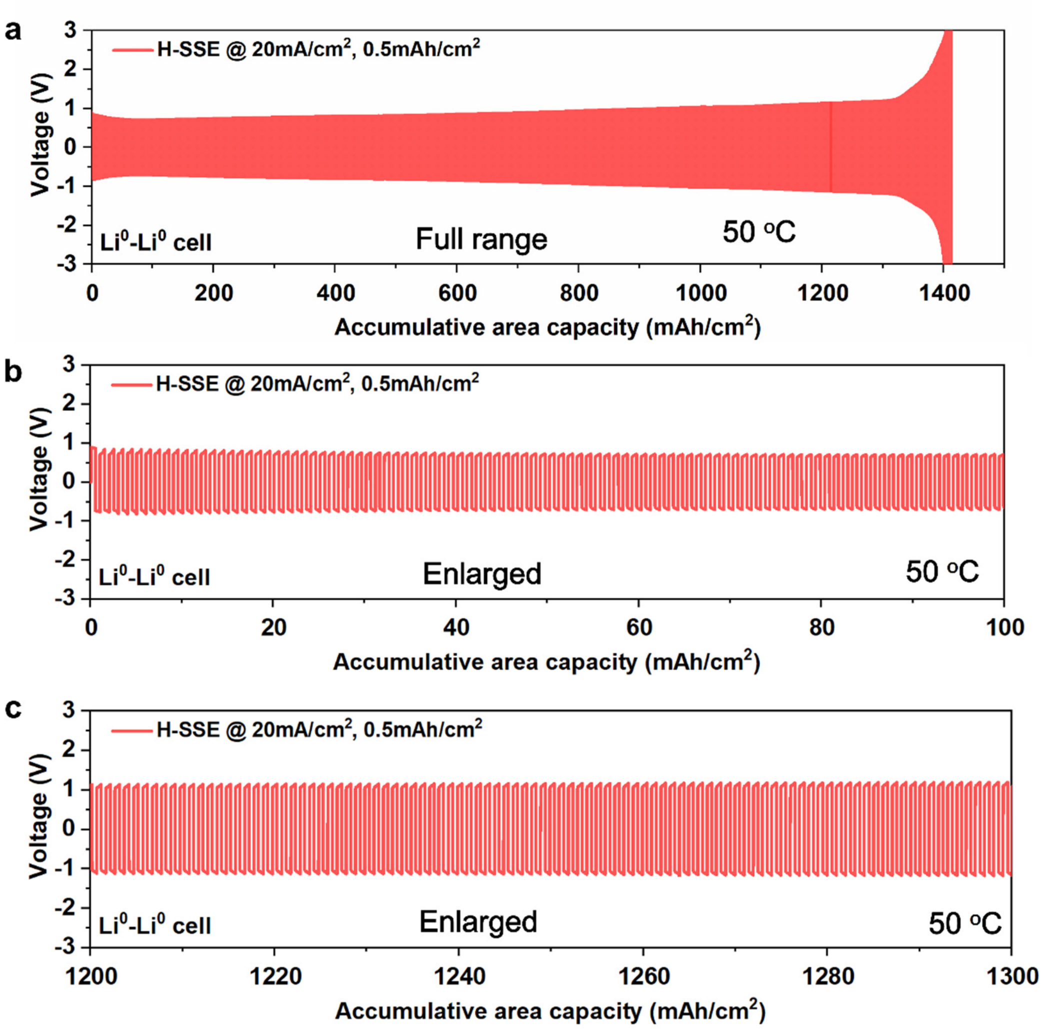

Figure S27. a, Durability of Li^0|H-SSE|Li^0 cells at **20mA/cm², 0.5mAh/cm². b, c**, Supplementary enlarged profiles. The cell was rested for 0.1s after charging and discharging for 1.5 min.

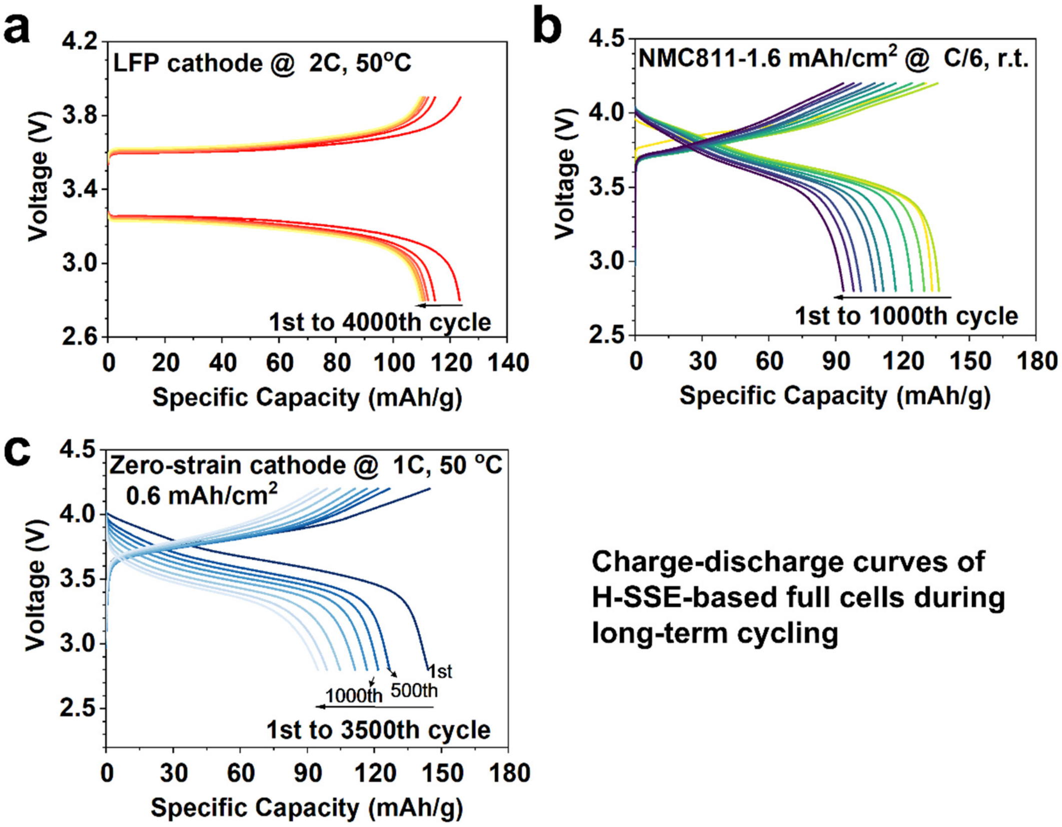

Figure S28. Charge-discharge curves of H-SSE based full cells when employing LFP **(a)**, NMC811 **(b)**, and a high-Ni, zero-Co, zero-strain cathode **(c)**.

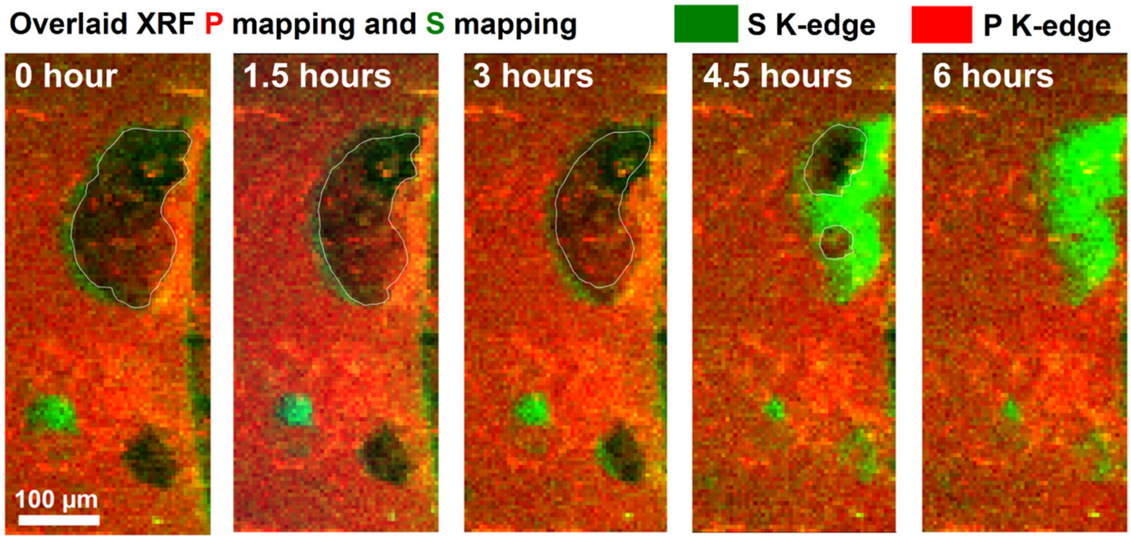

Figure S29. Overlaid S K-edge and P K-edge XRF mappings of the PCE-based *in-situ* cell when cycling at 0.05 mA/cm^2 for 6 hours.

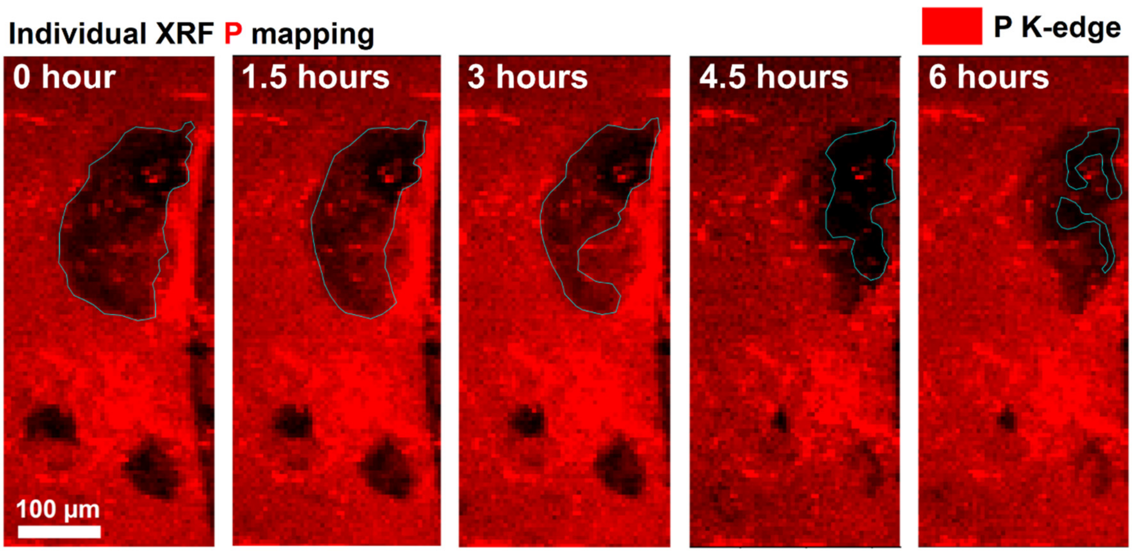

Figure S30. Individual P K-edge XRF mappings of the PCE-based *in-situ* cell when cycling at 0.05 mA/cm^2 for 6 hours.

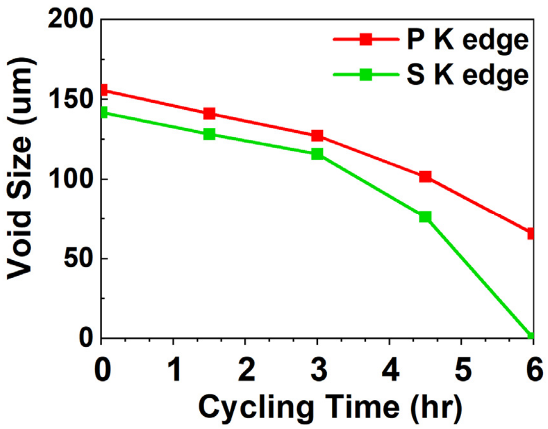

Figure S31. Size evolution of the 150-μm-sized void when cycling the *in-situ* cell at 0.05 mA/cm^2.

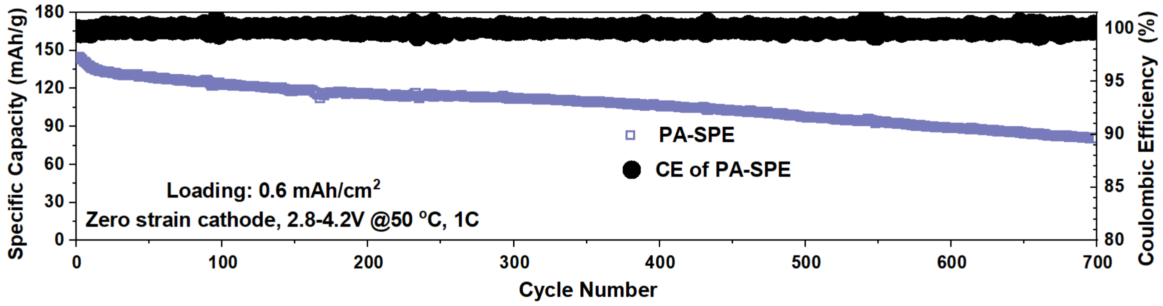

Figure S32. Performance of PA-SPE-based full cells when employing the high-Ni, zero-Co, zero-strain cathode. The left y-axis displays the discharge capacity, while the right y-axis represents the Coulombic efficiency of the cell.

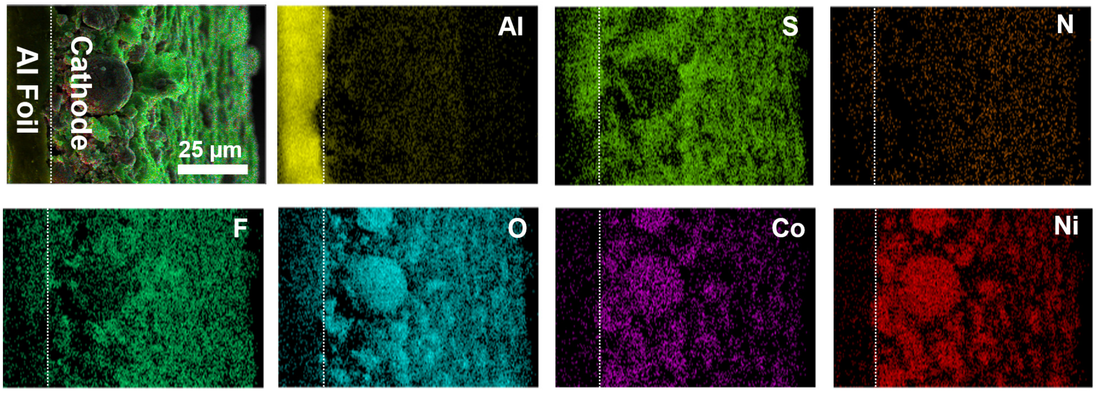

Figure S33. SEM-EDS mapping of the cross-section of the NMC811 cathode after cycling with H-SSE. The homogeneous distribution of N and S elements within the cathode indicates successful infiltration of lithium salt (LiTFSI) and succinonitrile solid crystal plasticizer into the cathode pores, thereby creating an ion conduction pathway.

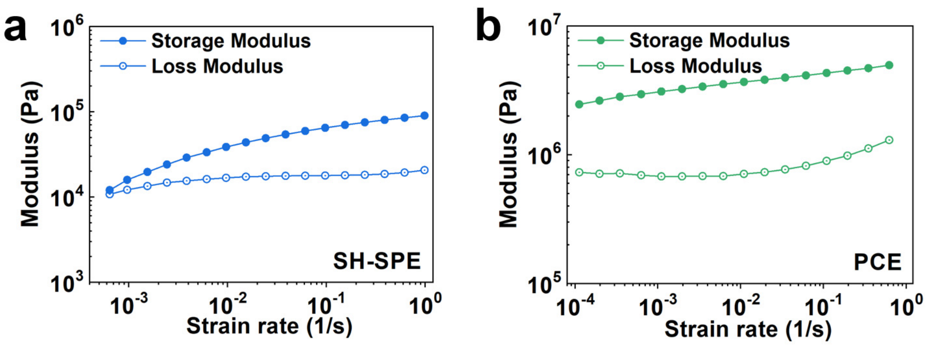

Figure S34. Viscoelasticity of SH-SPE **(a)** and PCE **(b)** showing the storage modulus (G') and loss modulus (G'') with increasing strain rate.

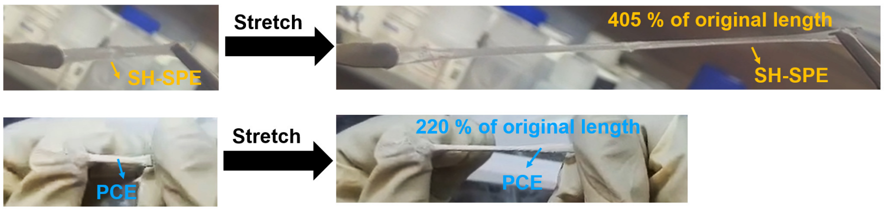

Figure S35. Photographs showing the stretchability of SH-SPE and PCE.

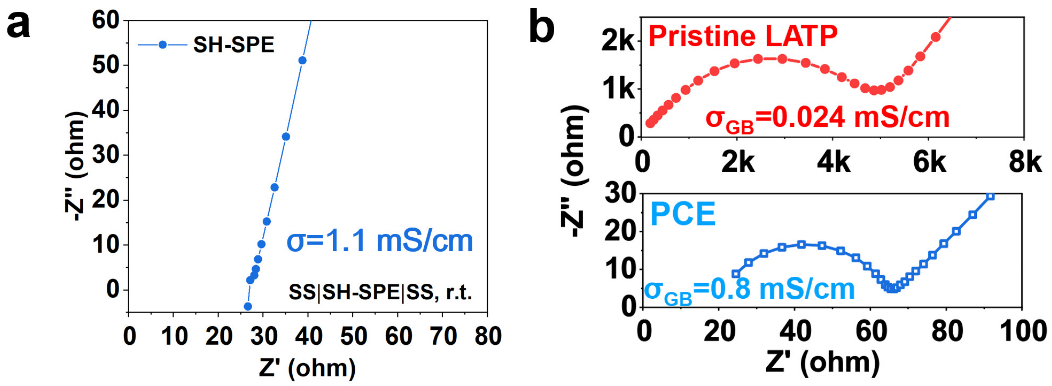

Figure S36. a, EIS plot of SS|SH-SPE|SS showing ionic conductivity of SH-SPE. SS refers to stainless steel blocking electrodes. **b,** EIS plots of SS|LATP|SS and SS|PCE|SS cells showing the grain boundary resistance and ionic conductivity.

Note: The differences in ionic conductivity between phases could cause Li^+ to preferentially transport through the more conductive phase, resulting in higher local current density and leading to non-uniform lithium deposition. In this study, the pristine SH-SPE exhibits a room temperature conductivity of 1.1 mS/cm, while the pristine LATP has a lower room temperature conductivity of 0.024 mS/cm due to its high grain boundary resistance. However, the infiltration of SH-SPE into the grain boundaries of LATP (Figure 1d) significantly enhances Li^+ conduction between LATP particles, reducing the grain boundary resistance to 55 ohm·cm^2, corresponding to a grain boundary conductivity of 0.8 mS/cm. Thus, in the PCE, both the polymer phase and ceramic phase show comparable conductivities of 1.1 mS/cm and 0.8 mS/cm, respectively.

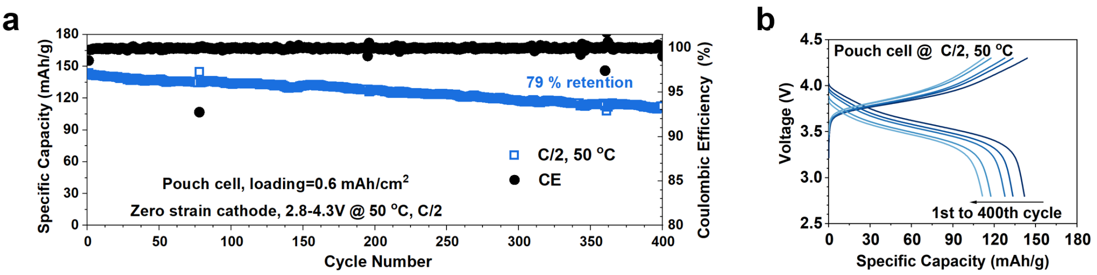

Figure S37. a, Performance of H-SSE when paired with a zero-strain cathode in pouch cells. The effective area of the zero-strain cathode is 7.8 cm^2, with a mass loading of 3.06 mg/cm^2. The cell was cycled at C/2 and 50 °C, with cut-off voltage of 2.8-4.3 V. **b,** Charge-discharge profiles of pouch cell when cycling at C/2 and 50 °C.

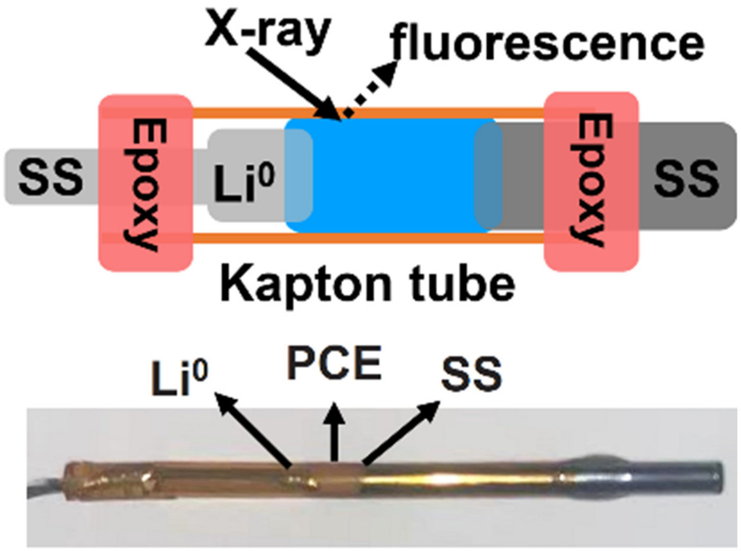

Figure S38. A schematic illustration of the tube battery and experimental set-up for XRF and XAS characterization.

Table S1. Comparison of the Li^0-Li^0 symmetric cell durability and full cell performance with previous Li^0-anode batteries on PEO-based solid polymer electrolytes.

a) Critical current density; b) 3V-class cathode: $LiFePO_4$; 4V-class cathode: NMC, $LiCoO_2$, etc.

**References**	**CCD [a)] (mA/cm^2)**	**Li^0-Li^0 cell Current/life/temp.**	**Current× cycle life (mAh/cm^2)**	**Cathode [b)]**	**Current Density**	**capacity/cycle life/retention**	**Loading (mg/cm^2)**	**Loading (mAh/cm^2) ×cycle number**
This work	30	1/2900hr/r.t.	**2900**	3 V	C/2	127/2400/84%	4.5	**1836**
		2/1000hr/50°C	**2000**	3 V	2C	123/4000/88%	2.3	**1564**
		5/300hr/50°C	**1500**	4 V	1C	144/2860/80%	3	**1844**
		10/150hr/50°C	**1500**	4V	C/6	130/1000/71%	7.4	**1600**
2024 Nat. Energy [1]	3.7	0.5/1000hr/	**500**	4V	C/2	210/400/80%	5.3	**460**
2024 Nat. Nanotechnol. [2]	NA	0.1/1000hr/60°C	**100**	3 V	0.5C	138/200/90%	1.5	**51**
				3 V	0.07C	130/35/95%	6.8	**41**
2024 Nat. Commun. [3]	0.5	0.1/2250hr/90°C	**225**	3 V	0.5C	160/1000/78%	1.6	**270**
2024 Nat. Mater. [4]	NA	0.05/1500hr/30°C	**75**	3 V	0.05C	147/40/92%	8.8	**53**
2024 Angew. Chem. [5]	1.1	0.4/1500hr/60°C	**600**	3V	2C	123/1500/80%	2	**510**
2024 Energy Environ. Sci. [6]	NA	0.1/100hr/25°C	**10**	3 V	0.1C	147/100/88%	1.2	**21**
				4 V	0.1C	152/100/66%	1.2	**26**
2024 Nano Energy [7]	0.5	0.1/1600hr/NA	**160**	4 V	0.1C	166/250/92%	3	**161**
		0.2/400hr/NA	**80**	4 V	NA	178/100/82%	10	**215**
2024 ACS Energy Lett. [8]	NA	0.4/500hr/70°C	**200**	3 V	2C	130/1000/76%	2.5	**425**
2023 J. Am. Chem. Soc. [9]	NA	0.1/400hr/25°C	**40**	3 V	0.5C	130/250/96%	2.5	**106**
				4 V	0.1C	134/100/84%	2.5	**42.5**
2023 Angew. Chem. [10]	0.3	0.1/1000hr/60°C	**100**	3 V	0.2C	149/600/75%	1.2	**122**
2023 PNAS [11]	2	0.5/700hr/25°C	**350**	3 V	0.5C	135/300/92%	2.5	**117**
				3 V	0.2C	160/100/80%	8	**136**
2023 Nano Energy [12]	NA	0.1/2000hr/60°C	**200**	3 V	1C	127/300/99%	1	**51**
2023 Nano Energy [13]	NA	0.5/600hr/NA	**300**	4 V	0.5C	145/100/92%	2.2	**28**
2023 Nano Energy [14]	0.7	0.1/900hr/25°C	**90**	3 V	0.2C	155/330/95%	1.7	**95**
				4 V	0.1C	152/100/83%	1.1	**24**
2023 Nano Energy [15]	0.2	0.1/320hr/60°C	**32**	3 V	0.1C	148/100/93%	NA	**NA**
2023 Adv. Energy Mater. [16]	NA	0.3/500hr/60°C	**150**	3 V	1C	147/300/99.7%	2	**102**
2022 Joule [17]	NA	0.75/1300hr/r.t.	**974**	3 V	0.25C	160/100/97%	4	**68**
2022 ACS Energy Lett. [18]	NA	0.1/445hr/70°C	**45**	4 V	C/20	160/80/80%	4.7	**80**
2022 Nano Energy [19]	NA	0.2/2400hr/60°C	**480**	3 V	0.5C	133/1350/83%	3.9	**895**
2022 Adv. Energy Mater. [20]	0.9	0.5/300hr/60°C	**150**	3 V	0.5C	160/500/70%	3	**255**
2022 Energy Environ. Sci. [21]	NA	0.2/2200hr/NA	**440**	4 V	0.5C	188/1000/80%	2.5	**537**
2022 Nat. Commun. [22]	NA	0.1/3800hr/90°C	**380**	3 V	0.5C	158/400/98%	1.5	**102**
				4 V	0.5C	145/100/87%	1.5	**25.2**

2022 Nat. Commun. [23]	1.2	0.1/1200hr/NA	**120**	3 V	0.25C	167/1200/87%	2	**480**
		1.2/120hr/NA	**144**					
2022 J. Am. Chem. Soc. [24]	NA	0.1/500hr/70°C	**50**	3 V	C/3	151/200/86%	4	**136**
2021 J. Am. Chem. Soc. [25]	2	0.4/400hr/NA	**160**	3 V	0.15C	160/120/78%	5	**102**
		1.2/100hr/NA	**120**	4 V	0.24C	140/120/86%	3	**77.4**
2021 Adv. Mater. [26]	NA	0.1/1200hr/NA	**120**	4 V	0.1C	177/100/78%	21	**450**
		1/1100hr/NA	**1100**	4 V	0.1C	163/100/89%	23	**450**
2021 Nano Lett. [27]	NA	0.1/300hr/60°C	**30**	3 V	1C	146/1000/84%	1	**170**
2020 Adv. Mater. [28]	0.5	0.1/1300hr/NA	**130**	3 V	0.5C	144/500/86%	2.4	**204**
2020 Energy Environ. Sci. [29]	NA	0.2/2500hr/NA	**500**	3 V	C/3	160/210/97%	3.3	**118**
				4 V	0.1C	135/110/90%	3.5	**82**
2020 Nano Lett. [30]	NA	0.1/300hr/60°C	**30**	3 V	0.2C	140/300/95%	1.5	**76.5**
2020 Nano Lett. [31]	NA	0.05/600hr/25°C	**30**	3 V	0.1C	166/250/77%	5	**212**
2020 Nano Lett. [32]	NA	1/560hr/90°C	**560**	3 V	1C	140/200/86%	2.5	**85**
		2/260hr/90°C	**520**	4 V	C/20	160/50/80%	2.5	**27**

Table S2. Comparison of the Li^0-Li^0 symmetric cell durability and full cell performance with previous Li^0-anode batteries on beyond-PEO solid polymer electrolytes.

a) Critical current density; b) 3V-class cathode: $LiFePO_4$; 4V-class cathode: NMC, $LiCoO_2$, etc.

References	CCD [a] (mA/cm^2)	Li^0-Li^0 cell Current/life/temp.	Current× cycle life (mAh/cm^2)	Cathode [b]	Current Density	capacity/cycle life/retention	Loading (mg/cm^2)	Loading (mAh/cm^2) ×cycle number
This work	30	1/2900hr/r.t.	**2900**	3 V	C/2	127/2400/84%	4.5	**1836**
		2/1000hr/50°C	**2000**	3 V	2C	123/4000/88%	2.3	**1564**
		5/300hr/50°C	**1500**	4 V	1C	144/2860/80%	3	**1844**
		10/150hr/50°C	**1500**	4 V	C/6	130/1000/71%	7.4	**1600**
2024 Nano Lett. [33]	0.5	0.1/1500hr/60°C	**150**	3 V	0.2C	160/300/99%	1.9	**97**
		0.5/400hr/60°C	**200**	4 V	0.2C	150/1/NA	1.9	**0.4**
2024 Angew. Chem. [34]	1	0.1/200hr/NA	**20**	3 V	0.5C	140/500/70%	1.5	**128**
				4 V	0.2C	271/15/99%	11	**45**
2023 Nat. Commun. [35]	NA	0.1/2500hr/NA	**250**	4 V	0.5C	164/200/89%	0.8	**34.4**
2023 Angew. Chem. [36]	NA	0.1/500hr/60°C	**50**	3 V	0.1C	150/440/99%	2.5	**187**
2023 Angew. Chem. [37]	NA	0.1/1000hr/60°C	**100**	4 V	0.1C	175/200/70%	3	**129**
2023 Angew. Chem. [38]	NA	0.1/1400hr/60°C	**140**	3 V	1C	137/350/97%	2	**119**
		0.2/800hr/60°C	**160**	3 V	2C	114/700/80%	2	**238**
2023 Adv. Mater. [39]	NA	0.5/700hr/NA	**350**	4 V	0.5C	170/700/84%	1.8	**270**
2023 Adv. Mater. [40]	NA	1/1000hr/NA	**1000**	3 V	2C	128/600/92%	1.2	**122**
2023 ACS Energy Lett. [41]	0.5	0.05/2000hr/40°C	**100**	4 V	2C	140/300/84%	2.3	**148**
				4 V	0.2C	160/100/86%	10.6	**228**

2022 Nature [42]	NA	10/1500hr/r.t.	**15000**	3 V	1C	93/1000/95%	1.5	**260**
				4 V	0.3C	110/100/88%	10	**213**
2022 Angew. Chem. [43]	NA	0.5/300hr/45°C	**150**	3 V	0.15C	159/350/82%	3.2	**190**
2022 Adv. Mater. [44]	2.4	0.1/4000hr/NA	**400**	3 V	0.4C	160/300/96%	1.5	**76.5**
2022 Energy Environ. Sci. [45]	NA	0.5/2200hr/NA	**1100**	3 V	0.5C	122/1200/99%	1	**204**
		1/800hr/NA	**800**	4 V	2C	160/200/82%	4	**172**
2022 Nano Energy [46]	NA	0.5/800hr/NA	**400**	4 V	1C	150/100/89%	2	**32**
2022 Nano Energy [47]	2	0.1/2200hr/NA	**220**	4 V	2C	133/1500/74%	2	**645**
2022 Nano Energy [48]	2.4	0.2/3000hr/NA	**600**	3 V	2C	140/200/67%	2	**68**
				4 V	0.2C	120/150/75%	4	**129**
2022 Nano Energy [49]	BA	0.5/1200hr/NA	**600**	4 V	1C	128/300/99%	4.6	**297**
2022 Adv. Energy Mater. [50]	0.5	0.3/550hr/NA	**165**	4 V	2C	130/300/90%	2.3	**148**
2021 Nat. Mater. [51]	1	0.2/2000hr/r.t.	**400**	NA	NA	NA	NA	**NA**
2020 Adv. Energy Mater. [52]	NA	0.1/800hr/NA	**80**	4 V	0.5C	191/200/85%	1.5	**64**

Table S3. Comparison of the Li^0-Li^0 symmetric cell durability and full cell performance with previous Li^0-anode batteries on self-healing solid polymer electrolytes.

a) Critical current density; b) 3V-class cathode: $LiFePO_4$; 4V-class cathode: NMC, $LiCoO_2$, etc.

**References**	**CCD [a)] (mA/cm^2)**	**Li^0-Li^0 cell Current/life/temp.**	**Current× cycle life (mAh/cm^2)**	**Cathode [b)]**	**Current Density**	**capacity/cycle life/retention**	**Loading (mg/cm^2)**	**Loading (mAh/cm^2) ×cycle number**
This work	30	1/2900hr/r.t.	**2900**	3 V	C/2	127/2400/84%	4.5	**1836**
		2/1000hr/50°C	**2000**	3 V	2C	123/4000/88%	2.3	**1564**
		5/300hr/50°C	**1500**	4 V	1C	144/2860/80%	3	**1844**
		10/150hr/50°C	**1500**	4V	C/6	130/1000/71%	7.4	**1600**
2024 Nat. Commun. [53]	NA	0.2/6000hr/60°C	**300**	3 V (SPAN)	0.3C	602/400/99.7%	2.1	**506**
2024 Adv. Funct. Mater. [54]	0.5	0.2/2100hr/NA	**105**	3 V	0.3C	157/480/80%	6	**490**
				4 V	0.3C	167/330/85%	1.5	**106**
2023 Angew. Chem. [10]	0.3	0.1/1000hr/60°C	**100**	3 V	0.2C	149/600/75%	1.2	**122**
2023 Mater. Horiz. [55]	NA	0.05/1600hr/r.t.	**80**	3 V	0.5C	134/400/68%	2.8	**190**
2023 ACS Appl. Energy Mater. [56]	1	1/20hr/25°C	**20**	NA	NA	NA	NA	**NA**
2023 Materials Today Energy [57]	NA	0.05/1400hr/r.t.	**70**	3 V	0.2C	150/300/94%	1.4	**71**
2023 Adv. Funct. Mater. [58]	NA	0.15/1300hr/NA	**195**	3 V	0.5C	151/230/98%	3	**117**
				4 V	0.3C	186/120/80%	3	**77**
2022 Energy Storage Materials [59]	0.1	0.05/500hr/60°C	**25**	3 V	0.1C	160/100/92%	NA	**NA**
2022 Nano Energy [49]	NA	0.5/1200hr/NA	**600**	4 V	1C	128/300/99%	4.6	**297**
2020 Angew. Chem. [60]	NA	0.2/600hr/NA	**120**	4 V	0.1C	115/200/86%	1.5	**64**

2020 ACS Energy Lett. [61]	NA	0.1/700hr/NA	**70**	4 V	0.1C	112/120/80%	1.2	**31**
2020 ACS Macro Lett. [62]	0.1	0.05/200hr/60°C	**10**	3 V	0.1C	145/70/79%	NA	**NA**
2019 J. Am. Chem. Soc. [63]	NA	0.015/0.44hr/60°C	**0.006**	NA	NA	NA	NA	**NA**

Supplementary References

1. Zhang, W., Koverga, V., Liu, S., Zhou, J., Wang, J., Bai, P., Tan, S., Dandu, N.K., Wang, Z., Chen, F., et al. (2024). Single-phase local-high-concentration solid polymer electrolytes for lithium-metal batteries. Nature Energy *9*, 386-400. 10.1038/s41560-023-01443-0.
2. Wan, J., Xie, J., Kong, X., Liu, Z., Liu, K., Shi, F., Pei, A., Chen, H., Chen, W., Chen, J., et al. (2019). Ultrathin, flexible, solid polymer composite electrolyte enabled with aligned nanoporous host for lithium batteries. Nat Nanotechnol *14*, 705-711. 10.1038/s41565-019-0465-3.
3. Zhu, G.R., Zhang, Q., Liu, Q.S., Bai, Q.Y., Quan, Y.Z., Gao, Y., Wu, G., and Wang, Y.Z. (2023). Non-flammable solvent-free liquid polymer electrolyte for lithium metal batteries. Nat Commun *14*, 4617. 10.1038/s41467-023-40394-8.
4. Han, S., Wen, P., Wang, H., Zhou, Y., Gu, Y., Zhang, L., Shao-Horn, Y., Lin, X., and Chen, M. (2023). Sequencing polymers to enable solid-state lithium batteries. Nat Mater *22*, 1515-1522. 10.1038/s41563-023-01693-z.
5. Cheng, Y., Cai, Z., Xu, J., Sun, Z., Wu, X., Han, J., Wang, Y.H., and Wang, M.S. (2024). Zwitterionic Cellulose-Based Polymer Electrolyte Enabled by Aqueous Solution Casting for High-Performance Solid-State Batteries. Angew Chem Int Ed Engl *63*, e202400477. 10.1002/anie.202400477.
6. Li, R., Hua, H., Yang, X., Tian, J., Chen, Q., Huang, R., Li, X., Zhang, P., and Zhao, J. (2024). The deconstruction of a polymeric solvation cage: a critical promotion strategy for PEO-based all-solid polymer electrolytes. Energy & Environmental Science *17*, 5601-5612. 10.1039/d4ee01188k.
7. Gong, Y., Wang, C., Xin, M., Chen, S., Xu, P., Li, D., Liu, J., Wang, Y., Xie, H., Sun, X., and Liu, Y. (2024). Ultra-thin and high-voltage-stable Bi-phasic solid polymer electrolytes for high-energy-density Li metal batteries. Nano Energy *119*. 10.1016/j.nanoen.2023.109054.
8. Guo, K., Li, S., Wang, J., Shi, Z., Wang, Y., and Xue, Z. (2024). In Situ Orthogonal Polymerization for Constructing Fast-Charging and Long-Lifespan Li Metal Batteries with Topological Copolymer Electrolytes. ACS Energy Letters *9*, 843-852. 10.1021/acsenergylett.3c02422.
9. Ding, P., Wu, L., Lin, Z., Lou, C., Tang, M., Guo, X., Guo, H., Wang, Y., and Yu, H. (2023). Molecular Self-Assembled Ether-Based Polyrotaxane Solid Electrolyte for Lithium Metal Batteries. J Am Chem Soc *145*, 1548-1556. 10.1021/jacs.2c06512.
10. Chen, J., Deng, X., Gao, Y., Zhao, Y., Kong, X., Rong, Q., Xiong, J., Yu, D., and Ding, S. (2023). Multiple Dynamic Bonds-Driven Integrated Cathode/Polymer Electrolyte for Stable All-Solid-State Lithium Metal Batteries. Angew Chem Int Ed Engl *62*, e202307255. 10.1002/anie.202307255.
11. Li, C., Hu, A., Zhang, X., Ni, H., Fan, J., Yuan, R., Zheng, M., and Dong, Q. (2023). An intrinsic polymer electrolyte via in situ cross-linked for solid lithium-based batteries with high performance. PNAS Nexus *2*, pgad263. 10.1093/pnasnexus/pgad263.
12. Han, L., Liu, Y., Liao, C., Zhao, Y., Cao, Y., Kan, Y., Zhu, J., and Hu, Y. (2023). Noncombustible 7 μm-thick solid polymer electrolyte for highly energy density solid state lithium batteries. Nano Energy *112*. 10.1016/j.nanoen.2023.108448.
13. Saleem, A., Iqbal, R., Majeed, M.K., Hussain, A., Akbar, A.R., Hussain, Z., Jabar, B., Rauf, S., and Shaw, L.L. (2024). Boosting lithium-ion conductivity of polymer electrolyte by selective introduction of covalent organic frameworks for safe lithium metal batteries. Nano Energy *128*. 10.1016/j.nanoen.2024.109848.
14. Zheng, J., Liu, S., Huang, H., Zhou, H., Li, H., Li, L., Jiang, G., Zhang, H., Geng, X., An, B., and Sun, C. (2023). Heterocyclic polymer supported cathode/Li interface layers to lower the operational temperature of PEO-based Li-batteries. Nano Energy *118*. 10.1016/j.nanoen.2023.108975.
15. Chen, Z., Jia, H., Yan, S., and Gohy, J.-F. (2023). Polymer-coated silica dual functional fillers to improve the performance of poly(ethylene oxide)-based solid electrolytes. Nano Energy *114*. 10.1016/j.nanoen.2023.108637.
16. Kim, E., Jamal, H., Jeon, I., Khan, F., Chun, S.E., and Kim, J.H. (2023). Functionality of 1-Butyl-2,3-Dimethylimidazolium Bromide (BMI-Br) as a Solid Plasticizer in PEO-Based Polymer Electrolyte for Highly Reliable Lithium Metal Batteries. Advanced Energy Materials *13*. 10.1002/aenm.202301674.
17. Cheng, Q., Jin, T., Miao, Y., Liu, Z., Borovilas, J., Zhang, H., Liu, S., Kim, S.-Y., Zhang, R., Wang, H., et al. (2022). Stabilizing lithium plating in polymer electrolytes by concentration-polarization-induced phase transformation. Joule *6*, 2372-2389. 10.1016/j.joule.2022.08.001.
18. Arrese-Igor, M., Martinez-Ibañez, M., Pavlenko, E., Forsyth, M., Zhu, H., Armand, M., Aguesse, F., and López-Aranguren, P. (2022). Toward High-Voltage Solid-State Li-Metal Batteries with Double-Layer Polymer Electrolytes. ACS Energy Letters *7*, 1473-1480. 10.1021/acsenergylett.2c00488.
19. Yang, L., Nie, Y., Liu, Y., Zheng, Y., Luo, D., Yang, N., Ma, Q., Xu, M., Ma, X., Yu, A., et al. (2022). The plasticizer-free composite block copolymer electrolytes for ultralong lifespan all-solid-state lithium-metal batteries. Nano Energy *100*. 10.1016/j.nanoen.2022.107499.
20. Ma, Y., Wan, J., Yang, Y., Ye, Y., Xiao, X., Boyle, D.T., Burke, W., Huang, Z., Chen, H., Cui, Y., et al. (2022). Scalable, Ultrathin, and High-Temperature-Resistant Solid Polymer Electrolytes for Energy-Dense Lithium Metal Batteries. Advanced Energy Materials *12*. 10.1002/aenm.202103720.
21. Wang, H., Song, J., Zhang, K., Fang, Q., Zuo, Y., Yang, T., Yang, Y., Gao, C., Wang, X., Pang, Q., and Xia, D. (2022).

A strongly complexed solid polymer electrolyte enables a stable solid state high-voltage lithium metal battery. Energy & Environmental Science *15*, 5149-5158. 10.1039/d2ee02904a.

22. Su, Y., Rong, X., Gao, A., Liu, Y., Li, J., Mao, M., Qi, X., Chai, G., Zhang, Q., Suo, L., et al. (2022). Rational design of a topological polymeric solid electrolyte for high-performance all-solid-state alkali metal batteries. Nat Commun *13*, 4181. 10.1038/s41467-022-31792-5.
23. Hu, J., Lai, C., Chen, K., Wu, Q., Gu, Y., Wu, C., and Li, C. (2022). Dual fluorination of polymer electrolyte and conversion-type cathode for high-capacity all-solid-state lithium metal batteries. Nat Commun *13*, 7914. 10.1038/s41467-022-35636-0.
24. Qiao, L., Rodriguez Pena, S., Martinez-Ibanez, M., Santiago, A., Aldalur, I., Lobato, E., Sanchez-Diez, E., Zhang, Y., Manzano, H., Zhu, H., et al. (2022). Anion pi-pi Stacking for Improved Lithium Transport in Polymer Electrolytes. J Am Chem Soc *144*, 9806-9816. 10.1021/jacs.2c02260.
25. Xu, B., Li, X., Yang, C., Li, Y., Grundish, N.S., Chien, P.H., Dong, K., Manke, I., Fang, R., Wu, N., et al. (2021). Interfacial Chemistry Enables Stable Cycling of All-Solid-State Li Metal Batteries at High Current Densities. J Am Chem Soc *143*, 6542-6550. 10.1021/jacs.1c00752.
26. He, F., Tang, W., Zhang, X., Deng, L., and Luo, J. (2021). High Energy Density Solid State Lithium Metal Batteries Enabled by Sub-5 microm Solid Polymer Electrolytes. Adv Mater *33*, e2105329. 10.1002/adma.202105329.
27. Han, L., Liao, C., Mu, X., Wu, N., Xu, Z., Wang, J., Song, L., Kan, Y., and Hu, Y. (2021). Flame-Retardant ADP/PEO Solid Polymer Electrolyte for Dendrite-Free and Long-Life Lithium Battery by Generating Al, P-rich SEI Layer. Nano Lett *21*, 4447-4453. 10.1021/acs.nanolett.1c01137.
28. Wang, H., Wang, Q., Cao, X., He, Y., Wu, K., Yang, J., Zhou, H., Liu, W., and Sun, X. (2020). Thiol-Branched Solid Polymer Electrolyte Featuring High Strength, Toughness, and Lithium Ionic Conductivity for Lithium-Metal Batteries. Adv Mater *32*, e2001259. 10.1002/adma.202001259.
29. Yang, X., Jiang, M., Gao, X., Bao, D., Sun, Q., Holmes, N., Duan, H., Mukherjee, S., Adair, K., Zhao, C., et al. (2020). Determining the limiting factor of the electrochemical stability window for PEO-based solid polymer electrolytes: main chain or terminal –OH group? Energy & Environmental Science *13*, 1318-1325. 10.1039/d0ee00342e.
30. Cui, Y., Wan, J., Ye, Y., Liu, K., Chou, L.Y., and Cui, Y. (2020). A Fireproof, Lightweight, Polymer-Polymer Solid-State Electrolyte for Safe Lithium Batteries. Nano Lett *20*, 1686-1692. 10.1021/acs.nanolett.9b04815.
31. He, Y., Chen, S., Nie, L., Sun, Z., Wu, X., and Liu, W. (2020). Stereolithography Three-Dimensional Printing Solid Polymer Electrolytes for All-Solid-State Lithium Metal Batteries. Nano Lett *20*, 7136-7143. 10.1021/acs.nanolett.0c02457.
32. Li, X., Zheng, Y., Duan, Y., Shang, M., Niu, J., and Li, C.Y. (2020). Designing Comb-Chain Crosslinker-Based Solid Polymer Electrolytes for Additive-Free All-Solid-State Lithium Metal Batteries. Nano Lett *20*, 6914-6921. 10.1021/acs.nanolett.0c03033.
33. Ye, F., Wang, Z., Li, M., Zhang, J., Wang, D., Liu, M., Liu, A., Lin, H., Kim, H.T., and Wang, J. (2024). High-Entropy Polymer Electrolytes Derived from Multivalent Polymeric Ligands for Solid-State Lithium Metal Batteries with Accelerated Li(+) Transport. Nano Lett *24*, 6850-6857. 10.1021/acs.nanolett.4c00154.
34. Zhao, Y., Li, L., Zhou, D., Ma, Y., Zhang, Y., Yang, H., Fan, S., Tong, H., Li, S., and Qu, W. (2024). Opening and Constructing Stable Lithium-ion Channels within Polymer Electrolytes. Angew Chem Int Ed Engl *63*, e202404728. 10.1002/anie.202404728.
35. Tang, L., Chen, B., Zhang, Z., Ma, C., Chen, J., Huang, Y., Zhang, F., Dong, Q., Xue, G., Chen, D., et al. (2023). Polyfluorinated crosslinker-based solid polymer electrolytes for long-cycling 4.5 V lithium metal batteries. Nat Commun *14*, 2301. 10.1038/s41467-023-37997-6.
36. Zhou, H.Y., Ou, Y., Yan, S.S., Xie, J., Zhou, P., Wan, L., Xu, Z.A., Liu, F.X., Zhang, W.L., Xia, Y.C., and Liu, K. (2023). Supramolecular Polymer Ion Conductor with Weakened Li Ion Solvation Enables Room Temperature All-Solid-State Lithium Metal Batteries. Angew Chem Int Ed Engl *62*, e202306948. 10.1002/anie.202306948.
37. Xie, X., Wang, Z., He, S., Chen, K., Huang, Q., Zhang, P., Hao, S.M., Wang, J., and Zhou, W. (2023). Influencing Factors on Li-ion Conductivity and Interfacial Stability of Solid Polymer Electrolytes, Exampled by Polycarbonates, Polyoxalates and Polymalonates. Angew Chem Int Ed Engl *62*, e202218229. 10.1002/anie.202218229.
38. Zhao, Z., Zhou, X., Zhang, B., Huang, F., Wang, Y., Ma, Z., and Liu, J. (2023). Regulating Steric Hindrance of Porous Organic Polymers in Composite Solid-State Electrolytes to Induce the Formation of LiF-Rich SEI in Li-Ion Batteries. Angew Chem Int Ed Engl *62*, e202308738. 10.1002/anie.202308738.
39. Qi, S., Li, M., Gao, Y., Zhang, W., Liu, S., Zhao, J., and Du, L. (2023). Enabling Scalable Polymer Electrolyte with Dual-Reinforced Stable Interface for 4.5 V Lithium-Metal Batteries. Adv Mater *35*, e2304951. 10.1002/adma.202304951.
40. Mu, K., Wang, D., Dong, W., Liu, Q., Song, Z., Xu, W., Yao, P., Chen, Y., Yang, B., Li, C., et al. (2023). Hybrid Crosslinked Solid Polymer Electrolyte via In-Situ Solidification Enables High-Performance Solid-State Lithium Metal Batteries. Adv Mater *35*, e2304686. 10.1002/adma.202304686.
41. Dong, X., Mayer, A., Liu, X., Passerini, S., and Bresser, D. (2023). Single-Ion Conducting Multi-block Copolymer Electrolyte for Lithium-Metal Batteries with High Mass Loading NCM811 Cathodes. ACS Energy Letters *8*, 1114-1121. 10.1021/acsenergylett.2c02806.

42. Lee, M.J., Han, J., Lee, K., Lee, Y.J., Kim, B.G., Jung, K.N., Kim, B.J., and Lee, S.W. (2022). Elastomeric electrolytes for high-energy solid-state lithium batteries. Nature *601*, 217-222. 10.1038/s41586-021-04209-4.
43. Li, W., Gao, J., Tian, H., Li, X., He, S., Li, J., Wang, W., Li, L., Li, H., Qiu, J., and Zhou, W. (2022). SnF(2) -Catalyzed Formation of Polymerized Dioxolane as Solid Electrolyte and its Thermal Decomposition Behavior. Angew Chem Int Ed Engl *61*, e202114805. 10.1002/anie.202114805.
44. Su, Y., Rong, X., Li, H., Huang, X., Chen, L., Liu, B., and Hu, Y.S. (2023). High-Entropy Microdomain Interlocking Polymer Electrolytes for Advanced All-Solid-State Battery Chemistries. Adv Mater *35*, e2209402. 10.1002/adma.202209402.
45. Xiang, J., Zhang, Y., Zhang, B., Yuan, L., Liu, X., Cheng, Z., Yang, Y., Zhang, X., Li, Z., Shen, Y., et al. (2021). A flame-retardant polymer electrolyte for high performance lithium metal batteries with an expanded operation temperature. Energy & Environmental Science *14*, 3510-3521. 10.1039/d1ee00049g.
46. Lin, Z., Guo, X., Zhang, R., Tang, M., Ding, P., Zhang, Z., Wu, L., Wang, Y., Zhao, S., Zhang, Q., and Yu, H. (2022). Molecular structure adjustment enhanced anti-oxidation ability of polymer electrolyte for solid-state lithium metal battery. Nano Energy *98*. 10.1016/j.nanoen.2022.107330.
47. Chen, L., Gu, T., Ma, J., Yang, K., Shi, P., Biao, J., Mi, J., Liu, M., Lv, W., and He, Y.-B. (2022). In situ construction of Li3N-enriched interface enabling ultra-stable solid-state LiNi0.8Co0.1Mn0.1O2/lithium metal batteries. Nano Energy *100*. 10.1016/j.nanoen.2022.107470.
48. Li, J., Zhang, H., Cui, Y., Da, H., Cai, Y., and Zhang, S. (2022). Constructing interfacial gradient layers and enhancing lithium salt dissolution kinetics for high-rate solid-state batteries. Nano Energy *102*. 10.1016/j.nanoen.2022.107716.
49. Chang, C., Yao, Y., Li, R., Guo, Z.H., Li, L., Pan, C., Hu, W., and Pu, X. (2022). Self-healing single-ion-conductive artificial polymeric solid electrolyte interphases for stable lithium metal anodes. Nano Energy *93*. 10.1016/j.nanoen.2021.106871.
50. Liang, H.P., Zarrabeitia, M., Chen, Z., Jovanovic, S., Merz, S., Granwehr, J., Passerini, S., and Bresser, D. (2022). Polysiloxane-Based Single-Ion Conducting Polymer Blend Electrolyte Comprising Small-Molecule Organic Carbonates for High-Energy and High-Power Lithium-Metal Batteries. Advanced Energy Materials *12*. 10.1002/aenm.202200013.
51. Wang, Y., Zanelotti, C.J., Wang, X., Kerr, R., Jin, L., Kan, W.H., Dingemans, T.J., Forsyth, M., and Madsen, L.A. (2021). Solid-state rigid-rod polymer composite electrolytes with nanocrystalline lithium ion pathways. Nat Mater *20*, 1255-1263. 10.1038/s41563-021-00995-4.
52. Yu, X., Wang, L., Ma, J., Sun, X., Zhou, X., and Cui, G. (2020). Selectively Wetted Rigid–Flexible Coupling Polymer Electrolyte Enabling Superior Stability and Compatibility of High-Voltage Lithium Metal Batteries. Advanced Energy Materials *10*. 10.1002/aenm.201903939.
53. Pei, F., Wu, L., Zhang, Y., Liao, Y., Kang, Q., Han, Y., Zhang, H., Shen, Y., Xu, H., Li, Z., and Huang, Y. (2024). Interfacial self-healing polymer electrolytes for long-cycle solid-state lithium-sulfur batteries. Nat Commun *15*, 351. 10.1038/s41467-023-43467-w.
54. Wu, L., Pei, F., Cheng, D., Zhang, Y., Cheng, H., Huang, K., Yuan, L., Li, Z., Xu, H., and Huang, Y. (2023). Flame-Retardant Polyurethane-Based Solid-State Polymer Electrolytes Enabled by Covalent Bonding for Lithium Metal Batteries. Advanced Functional Materials *34*. 10.1002/adfm.202310084.
55. Lin, X., Xu, S., Tong, Y., Liu, X., Liu, Z., Li, P., Liu, R., Feng, X., Shi, L., and Ma, Y. (2023). A self-healing polymerized-ionic-liquid-based polymer electrolyte enables a long lifespan and dendrite-free solid-state Li metal batteries at room temperature. Mater Horiz *10*, 859-868. 10.1039/d2mh01289h.
56. Daniels, E.L., Runge, J.R., Oshinowo, M., Leese, H.S., and Buchard, A. (2023). Cross-Linking of Sugar-Derived Polyethers and Boronic Acids for Renewable, Self-Healing, and Single-Ion Conducting Organogel Polymer Electrolytes. ACS Appl Energy Mater *6*, 2924-2935. 10.1021/acsaem.2c03937.
57. Ling, C., Naren, T., Liu, X., Yang, J., Xiao, P., Wei, W., Ji, X., Kuang, G.-C., and Chen, L. (2023). In-situ polymerization induced phase separation to develop high-performance self-healable polymeric electrolytes for lithium metal battery. Materials Today Energy *36*. 10.1016/j.mtener.2023.101372.
58. Zhao, L., Du, Y., Zhao, E., Li, C., Sun, Z., Li, Y., and Li, H. (2023). Dynamic Supramolecular Polymer Electrolyte to Boost Ion Transport Kinetics and Interfacial Stability for Solid-State Batteries. Advanced Functional Materials *33*. 10.1002/adfm.202214881.
59. Huang, Y., Shi, Z., Wang, H., Wang, J., and Xue, Z. (2022). Shape-memory and self-healing polyurethane-based solid polymer electrolytes constructed from polycaprolactone segment and disulfide metathesis. Energy Storage Materials *51*, 1-10. 10.1016/j.ensm.2022.06.021.
60. Jaumaux, P., Liu, Q., Zhou, D., Xu, X., Wang, T., Wang, Y., Kang, F., Li, B., and Wang, G. (2020). Deep-Eutectic-Solvent-Based Self-Healing Polymer Electrolyte for Safe and Long-Life Lithium-Metal Batteries. Angew Chem Int Ed Engl *59*, 9134-9142. 10.1002/anie.202001793.
61. Liu, Q., Zhou, D., Shanmukaraj, D., Li, P., Kang, F., Li, B., Armand, M., and Wang, G. (2020). Self-Healing Janus Interfaces for High-Performance LAGP-Based Lithium Metal Batteries. ACS Energy Letters *5*, 1456-1464. 10.1021/acsenergylett.0c00542.
62. Zhou, B., Yang, M., Zuo, C., Chen, G., He, D., Zhou, X., Liu, C., Xie, X., and Xue, Z. (2020). Flexible, Self-Healing, and Fire-Resistant Polymer Electrolytes Fabricated via Photopolymerization for All-Solid-State Lithium Metal Batteries.

ACS Macro Lett *9*, 525-532. 10.1021/acsmacrolett.9b01024.
63. Jing, B.B., and Evans, C.M. (2019). Catalyst-Free Dynamic Networks for Recyclable, Self-Healing Solid Polymer Electrolytes. J Am Chem Soc *141*, 18932-18937. 10.1021/jacs.9b09811.
